# An integrate-and-fire approach to Ca^2+^ signaling. Part II: Cumulative refractoriness

**DOI:** 10.1016/j.bpj.2023.11.015

**Published:** 2023-11-19

**Authors:** Lukas Ramlow, Martin Falcke, Benjamin Lindner

**Affiliations:** 1 Bernstein Center for Computational Neuroscience Berlin, Berlin, Germany; 2 Department of Physics, Humboldt University Berlin, Berlin, Germany; 3 Max Delbrück Center for Molecular Medicine, Berlin, Germany

## Abstract

Inositol 1,4,5-trisphosphate-induced Ca^2+^ signaling is a second messenger system used by almost all eukaryotic cells. The agonist concentration stimulating Ca^2+^ signals is encoded in the frequency of a Ca^2+^ concentration spike sequence. When a cell is stimulated, the interspike intervals (ISIs) often show a distinct transient during which they gradually increase, a system property we refer to as cumulative refractoriness. We extend a previously published stochastic model to include the Ca^2+^ concentration in the intracellular Ca^2+^ store as a slow adaptation variable. This model can reproduce both stationary and transient statistics of experimentally observed ISI sequences. We derive approximate expressions for the mean and coefficient of variation of the stationary ISIs. We also consider the response to the onset of a constant stimulus and estimate the length of the transient and the strength of the adaptation of the ISI. We show that the adaptation sets the coefficient of variation in agreement with current ideas derived from experiments. Moreover, we explain why, despite a pronounced transient behavior, ISI correlations can be weak, as often observed in experiments. Finally, we fit our model to reproduce the transient statistics of experimentally observed ISI sequences in stimulated HEK cells. The fitted model is able to qualitatively reproduce the relationship between the stationary interval correlations and the number of transient intervals, as well as the strength of the ISI adaptation. We also find positive correlations in the experimental sequence that cannot be explained by our model.

## Significance

Intracellular Ca^2+^ is a universal second messenger that regulates many processes in different cell types through stimulus-dependent spiking patterns. The spike sequence exhibits an initial transient during which the interspike intervals gradually increase. After the transient, when spiking is stationary, the intervals are correlated. We follow the idea that both can be explained by a cumulative depletion and slow replenishment of the intracellular Ca^2+^ store—the endoplasmic reticulum. We propose a stochastic integrate-and-fire model with a slow variable that captures the Ca^2+^ concentration in the endoplasmic reticulum and ask what are the consequences of the cumulative depletion for the spiking statistics? We compare the model predictions with experimental spike sequences in stimulated HEK cells.

## Introduction

The inositol trisphosphate (IP_3_)-induced Ca^2+^ signaling pathway translates extracellular signals in the form of plasma membrane receptor agonist concentrations into intracellular responses by increasing the cytosolic Ca^2+^ concentration in a stimulus-dependent pattern (1,2,3,4,5,6). Repetitive sequences of Ca^2+^ spikes are used to regulate many processes in various cell types (1,4,7,8). The concentration increase can be caused either by Ca^2+^ entry from the extracellular medium through plasma membrane channels or by Ca^2+^ release from intracellular storage compartments. In the following, we will focus on IP_3_-induced Ca^2+^ release from the endoplasmic reticulum (ER), which is the predominant Ca^2+^ release mechanism in many cell types (9). IP_3_ sensitizes the IP_3_ receptor Ca^2+^ channels (IP_3_R) on the ER membrane for Ca^2+^ binding, such that Ca^2+^ released from the ER through one channel increases the open probability of neighboring channels (10,11). This positive feedback of Ca^2+^ on its own release is called Ca^2+^-induced Ca^2+^ release. It spreads local release to cell-wide Ca^2+^ spikes. The timing of Ca^2+^ spikes is random. Spike sequences exhibit a linear cumulant relation between mean and standard deviation of interspike intervals (ISIs) (4,12,13,14,15,16,17,18,19,20,21). Ca^2+^ released during a spike is removed from the cytosol either by sarcoendoplasmic reticulum Ca^2+^ ATPases (SERCAs) into the ER or by plasma membrane Ca^2+^ ATPases into extracellular space.

Many cell types exhibit an initial transient upon onset of stimulation, during which the ISI gradually increases and the spike amplitude gradually decreases until a stationary state is reached (12,22,23,24,25). The transient indicates a process that is slow compared with typical ISIs and cumulatively reduces the spike generation probability over multiple spike times. We would like to learn by modeling what information about this slow adaptive process and spike generation we can gain from the spike time statistics.

Lock and Parker report that partial depletion of the ER during the Ca^2+^ spike may contribute to the termination of Ca^2+^ release from the ER and thus to termination of spikes by a dependence of IP_3_R gating on lumenal Ca^2+^ (26,27). Suzuki et al. have shown that in some cells the Ca^2+^ concentration in the ER decreases cumulatively over several spikes (28). This can also be viewed as a slow decline in total Ca^2+^ in the cell and is likely the result of a net loss of Ca^2+^ during a spike and a slow replenishment by store-operated Ca^2+^ entry (SOCE) between spikes. We follow the idea that this cumulative depletion constitutes a slow process that may explain the observed transients in the sequence of ISIs. The importance of membrane fluxes controlling the total Ca^2+^ concentration for the regulation of Ca^2+^ spikes has been demonstrated experimentally (29,30,31,32) and by several deterministic models (33,34,35). These fluxes represent a slow feedback of spiking to the spike generation probability. Slow feedbacks have been identified as crucial determinants of ISI statistics and spike train information content (4,36,37,38,39,40). Hence, we investigate here the relation between slow ER dynamics and first- and second-order ISI statistics and draw conclusions about the corresponding cell parameters.

We have recently proposed a two-component model that focuses on Ca^2+^ spiking as a stochastic point process (41). The first component describes the activity of clusters of IP_3_R channels and the second component describes the dynamics of the cytosolic Ca^2+^ concentration. The cluster dynamics are given in terms of a cyclic Markov chain that captures the stochastic release of Ca^2+^ from the ER by the puffs. The cytosolic Ca^2+^ concentration is described in the integrate-and-fire framework and driven by the puff current. This model generates a *renewal* spike train without any initial transient. In this study, we extend the integrate-and-fire part of the model by a second variable that captures the partial depletion and slow replenishment of the ER. This system is mathematically very similar to an adaptive integrate-and-fire model. It generates a *nonrenewable* spike train and accounts for the experimentally observed transients in the ISI sequence.

Our paper is organized as follows. In the first section, we present the extended model and recall the kinetics of IP_3_ receptor clusters. In the second section, we consider how this additional slow adaptation-like variable affects the first- and second-order stationary statistics of the ISIs. We derive analytical expressions for the mean and coefficients of variation of the ISIs using a mean-adaptation approximation and discuss the observed correlation between intervals. In the following sections, we consider transient ISI statistics such as the length of the transient and strength of the adaptation of the ISIs. To estimate the length of the transient, we derive approximate expressions for the effective timescale on which the ER is depleted. We then ask how these transient statistics relate to the stationary interval correlations. Finally, we fit our model to reproduce experimentally observed ISI sequences and test whether the observed interval correlations and the observed relations between transient and stationary statistics can be reproduced by our model.

## Model

### Ca^2+^ store depletion and replenishment: An adaptive integrate-and-fire model

We extend our model from Part I (41) by a second variable $c_{\text{er}}$, associated with the Ca^2+^ concentration in the ER, that takes into account the depletion of the intracellular Ca^2+^ store upon firing of a Ca^2+^ spike. The extended IF part of the model reads as follows:

(1) $$\begin{array}{l} {\dot{c}}_{i}=-\left(c_{i}-c_{i}^{0}c_{\text{er}}\right)/\tau +j_{\text{puff}}\left(c_{i},c_{\text{er}}\right)\text{,}\\ {\dot{c}}_{\text{er}}=-\left(c_{\text{er}}-1\right)/\tau_{\text{er}}-\varepsilon c_{\text{er}}\sum_{i}\delta \left(t-t_{i}\right)\text{,}\\ \text{if}\;c_{i}\left(t\right)=c_{T}\to t_{i}=t\;\text{and}\;c_{i}\left(t\right)=c_{R}\text{.} \end{array}$$

In this model, which is derived in the Appendix subthreshold Ca2+ dynamics, $c_{i}={\left[{\text{Ca}}^{2+}\right]}_{i}/K_{\text{act}}$ is the nondimensional cytosolic Ca^2+^ concentration and $c_{\text{er}}={\left[{\text{Ca}}^{2+}\right]}_{\text{er}}/{\left[{\text{Ca}}^{2+}\right]}_{\text{er}}^{0}$ is the nondimensional ER Ca^2+^ concentration. Here, $K_{\text{act}}$ is the dissociation constant of the activating binding sites of the IP_3_R and ${\left[{\text{Ca}}^{2+}\right]}_{\text{er}}^{0}$ is the steady-state Ca^2+^ concentration in the ER. Whenever a spike is fired at a spike time $t_{i}$, the ER is partially depleted (second term of $c_{\text{er}}$-dynamics). In our model, $c_{\text{er}}\left(t\right)$ is immediately decreased by the net loss during a spike, $\varepsilon c_{\text{er}}\left(t_{i}^{-}\right)$ (the minus indicates an instant right *before* the spike time $t_{i}$). The delta kick results from the fact that a Ca^2+^ spike in the integrate-and-fire framework is only a point event in time (the spike shape is not described). The time right after a spike is denoted by $t_{i}^{+}$. Although we do not describe spikes by our model, remarks about what we consider to be a spike are necessary. A spike consists of a fast rise of the Ca^2+^ concentration $c_{i}$ in the whole cell, followed by a fast drop after the spike duration has passed. Spikes entail a global absolute refractory period without puffs in many cell types (42,43). Both spike and refractory period are global processes involving all clusters and are therefore less variable in duration than the spike generation process that we model. We allow for puff current immediately after a spike, i.e., at $t_{i}^{+}$ already. This implies that we consider the end of the absolute refractory period to be the end of the spike. In between spikes, the ER replenishes, and $c_{\text{er}}\left(t\right)$ returns exponentially to its steady-state value 1 with time constant $\tau_{\text{er}}$ (first term). To see a significant adaptation effect of the variable $c_{\text{er}}\left(t\right)$, its time constant $\tau_{\text{er}}$ should be larger than the mean ISI.

The first line in Eq. 1 is similar to the model in our previous paper and describes the dynamics of the cytosolic Ca^2+^ concentration $c_{i}$ with the additional fire-and-reset rule that whenever $c_{i}\left(t\right)=c_{T}$ a spike is said to be fired at $t_{i}=t$ and $c_{i}\left(t\right)$ is immediately reset to $c_{R}$. The first term on the r.h.s. is a deterministic linear current with timescale *τ*, which determines how fast $c_{i}\left(t\right)$ returns to the concentration $c_{i}^{0}c_{\text{er}}\left(t\right)$. This concentration would correspond to the stationary concentration if $c_{\text{er}}\left(t\right)$ was fixed and no puffs would occur. We set $c_{R}=c_{i}^{0}c_{\text{er}}\left(t_{i}^{+}\right)$, since we assume no puffs to occur in the absolute refractory period after the spike and $c_{\text{er}}\left(t\right)$ to change slowly compared with $c_{i}\left(t\right)$. This implies that $c_{i}\left(t\right)$ is not always reset to the same value.

The second term of the $c_{i}$-dynamics is the stochastic puff current, which captures the release of Ca^2+^ from the ER into the cytosol through randomly opening and closing clusters of IP_3_Rs:

(2) $$j_{\text{puff}}\left(c_{i},c_{\text{er}}\right)=pc_{\text{er}}\sum_{k=1}^{K}x_{k}\left(t\right)\text{.}$$

Here, *p* is a permeability-like parameter and $x_{k}\left(t\right)$ is the number of open channels in the *k*-th cluster. Cluster states are a Markov chain governed by a master equation

(3) $$\dot{p}=W\cdot p$$

with the transition rate matrix

**Figure:**
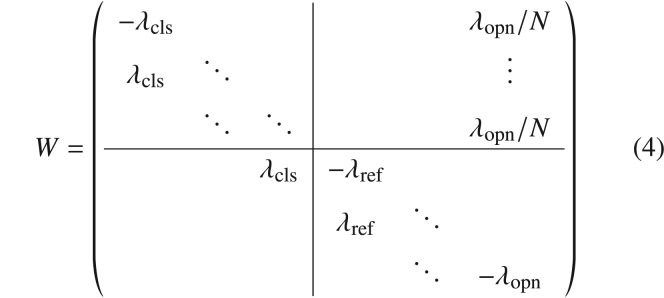

and probability vector

(5) $$p={\left(\begin{array}{cccccc} p\left(N\right) & \ldots & p\left(1\right) & p\left(0_{M}\right) & \ldots & p\left(0_{1}\right) \end{array}\right)}^{T}\text{.}$$

The state labels N…1 indicate open states and $0_{M}\ldots 0_{1}$ closed states (for more details see Part I, Fig. 2, and Eqs. 4–8 in (41)). In addition, the puff current depends linear on the concentration difference between ER and cytosol; the latter can be omitted because ${\left[{\text{Ca}}^{2+}\right]}_{\text{er}}\gg {\left[{\text{Ca}}^{2+}\right]}_{i}$ (44,45,46).

For the following discussion, it is important to distinguish two firing regimes. In the mean-driven regime, the mean puff current is strong enough to bring $c_{i}$ to the firing threshold. This implies that, even in a proper deterministic limit (K→∞, p→0 with finite and nonvanishing pK=const.), the model would still generate spikes. In contrast, in the fluctuation-driven (excitable) regime the mean puff current would drive the cytosolic Ca^2+^ concentration to a fixed point $c_{i}^{*}<c_{T}$ below the firing threshold. Only by a random fluctuation in the puff current can the concentration cross the threshold and a spike be elicited (no spiking in the deterministic limit).

The transition between the two regimes occurs when the fixed point $c_{i}^{*}$ falls exactly on the firing threshold while the ER is still completely filled. Hence, the bifurcation condition of the extended model, separating the mean- and fluctuation-driven regimes, is the same as in the first part of this paper (Eq. 35 in (41)):

(6) $$p\tau =\frac{c_{T}-c_{i}^{0}}{K\mu_{x}\left(c_{T}\right)}\text{.}$$

## Results

**Figure 1:**
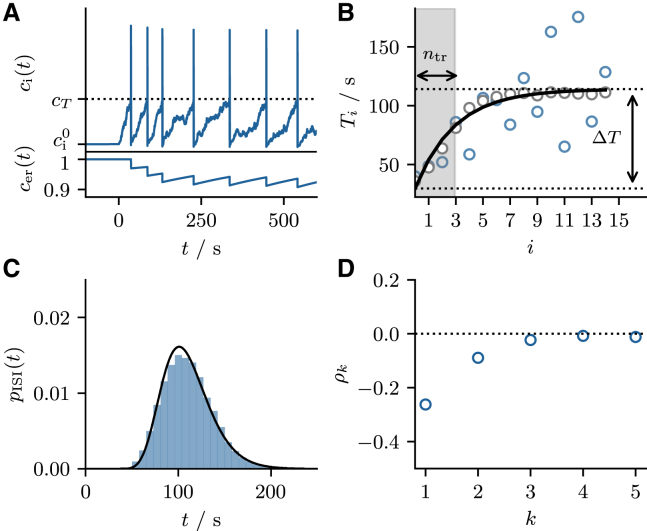
Cumulative refractoriness in a Ca^2+^ signaling model with store depletion. (*A*) The cytosolic and ER Ca^2+^ concentrations and $c_{\text{er}}\left(t\right)$ in response to a constant IP_3_ stimulation applied at t=0. The dotted line in (*A*) marks the firing threshold $c_{T}$. (*B*) The transient of the sequence of ISI $\left\{T_{i}\right\}$ (*blue circles*) and sequence of mean ISI $\left\{\left\langle T_{i}\right\rangle \right\}$ (*gray circles*). The black line shows a fit $T_{\infty }-\left(T_{\infty }-T_{0}\right)\exp\left(-i/n_{\text{tr}}\right)$ to the sequence of mean intervals from which the number of transient intervals $n_{\text{tr}}$ and cumulative refractory period $\Delta T=T_{\infty }-T_{0}$ are determined. The lower and upper dotted lines indicate the fit parameters $T_{0}$ and $T_{\infty }$, respectively. (*C*) The stationary ISI density together with an inverse Gaussian distribution with identical mean ⟨T⟩ and coefficient of variation $C_{V}$ of the ISI. (*D*) The serial correlation coefficient $\rho_{k}$, Eq. 23, as a function of the lag *k*. Parameters: τ=5s, p=0.015, $\tau_{\text{er}}=300\;s$, ε=0.03. To see this figure in color, go online.

We start with an illustration of the stochastic dynamics of the model obtained from numerical simulations of Eqs. 1, 2, and 3. The response of the two concentrations $c_{i}\left(t\right)$ and $c_{\text{er}}\left(t\right)$ to a constant IP_3_ stimulation applied at time $t_{0}=0$ are shown in Fig. 1 *A*. Before the stimulation ($t<t_{0}$), the concentrations rest at $c_{i}=c_{i}^{0}$ and $c_{\text{er}}=1$, all IP_3_R channels are closed, the puff current is zero, and there are no spikes generated in this state. Upon stimulation, the IP_3_R channels are activated and $c_{i}\left(t\right)$ begins to rise toward the threshold while $c_{\text{er}}\left(t\right)$ remains at 1. When the threshold is first reached, a spike is fired at time $t_{1}$, $c_{\text{er}}\left(t\right)$ is decreased by $\varepsilon c_{\text{er}}\left(t_{1}^{-}\right)$, and $c_{i}\left(t\right)$ is reset to $c_{R}=c_{i}^{0}c_{\text{er}}\left(t_{i}^{+}\right)$. The difference between the first spike time and the stimulation time defines the first interval, $T_{0}=t_{1}-t_{0}$ (not an ISI).

After the reset, $c_{i}\left(t\right)$ rises toward the threshold again and $c_{\text{er}}\left(t\right)$ is replenished slowly. During the transient intervals, the replenishment of $c_{\text{er}}\left(t\right)$ between two spikes is not sufficient to compensate for the depletion during a spike. This leads to a cumulative decrease of $c_{\text{er}}\left(t\right)$ (Fig. 1 *A*, *lower panel*) and, due to the resulting decrease of the puff current and of the reset value, also to a cumulative refractoriness in the sequence of ISIs $\left\{T_{i}\right\}$ (*blue circles* in Fig. 1 *B*). The gray circles in Fig. 1 *B* indicate the sequence of mean ISIs $\left\{\left\langle T_{i}\right\rangle \right\}$ obtained by averaging over many simulation trials. The latter sequence can be well-fitted by an exponential function

(7) $$T_{i}=T_{\infty }-\left(T_{\infty }-T_{0}\right)e^{-i/n_{\text{tr}}}$$

(*black line* in Fig. 1 *B*). The number of transient intervals $n_{\text{tr}}$ and the cumulative refractory period $\Delta T=T_{\infty }-T_{0}$ are the fit parameters. Replenishment and depletion balance on average after the transient, and the statistics of the ISIs no longer depend on the absolute time *t*. All ISIs follow the same probability density in this stationary state (*blue histogram* in Fig. 1 *C*), which can be well described by an inverse Gaussian (*black line*). Even in the stationary state, when intervals are identically distributed, they are not independent. The expected value of a certain interval $T_{i+1}$ depends on $c_{\text{er}}\left(t_{i}^{+}\right)$ at the beginning of that interval. Specifically, the smaller (larger) $c_{\text{er}}\left(t_{i}^{+}\right)$ the longer (shorter) $T_{i+1}$ on average. The value $c_{\text{er}}\left(t_{i}^{+}\right)$ in turn depends on the length of the previous interval $T_{i}$ in such a way that the longer (shorter) $T_{i}$ the larger (smaller) $c_{\text{er}}\left(t_{i}^{+}\right)$. As a result, the two intervals $T_{i}$ and $T_{i+1}$ (as well as the intervals $T_{i}$ and $T_{i+k}$) will in general be anticorrelated, as illustrated by means of the serial correlation coefficient (SCC) $\rho_{k}$ in Fig. 1 *D*.

### Stationary interspike interval statistics

The two-component model introduced above is difficult to treat analytically and numerically expensive. For this reason, we aim at a simplified description of Eqs. 1, 2, and 3 in terms of a Langevin equation, similar to what has been done in Part I of this paper for the renewal model. As mentioned above and shown in the first part of this paper, for $c_{\text{er}}=\text{const.}$ the stochastic puff current $j_{\text{puff}}\left(c_{i}\right)$ can be approximated by a Gaussian white noise with $c_{i}$-dependent mean $\mu \left(c_{i}\right)$ and intensity $D\left(c_{i}\right)$. This approximation relies on the rapid change of the number of open channels in a cluster $x_{k}\left(t\right)$ (41). In other words, we exploit that the correlation time of $x_{k}\left(t\right)$ is small compared with any other timescale in the system. Our model can then be approximated by the Langevin equation:

(8) $$\begin{array}{l} {\dot{c}}_{i}=-\left(c_{i}-c_{i}^{0}c_{\text{er}}\right)/\tau +\mu \left(c_{i},c_{\text{er}}\right)+\sqrt{2D\left(c_{i},c_{\text{er}}\right)}\xi \left(t\right)\\ {\dot{c}}_{\text{er}}=-\left(c_{\text{er}}-1\right)/\tau_{\text{er}}-\varepsilon c_{\text{er}}\sum_{i}\delta \left(t-t_{i}\right)\text{,}\\ \text{if}\;c_{i}\left(t\right)=c_{T}\to t_{i}=t\;\text{and}\;c_{i}\left(t\right)=c_{R}\text{.} \end{array}$$

Here, ξ(t) is a Gaussian white noise with correlation function $\left\langle \xi \left(t\right)\xi \left(t^{\prime }\right)\right\rangle =\delta \left(t-t^{\prime }\right)$ and we interpret the stochastic differential in the sense of Stratonovich (47,48). The mean $\mu \left(c_{i},c_{\text{er}}\right)=pc_{\text{er}}K\mu_{x}\left(c_{i}\right)$ and the noise intensity $D\left(c_{i},c_{\text{er}}\right)={\left(pc_{\text{er}}\right)}^{2}KD_{x}\left(c_{i}\right)$ of the puff current depend on $c_{i}\left(t\right)$ and on $c_{\text{er}}\left(t\right)$. The mean $\mu_{x}\left(c_{i}\right)$ and noise intensity $D_{x}\left(c_{i}\right)$ of a single cluster still depend solely on $c_{i}\left(t\right)$ through the $c_{i}$-dependence of the opening rate. Both $\mu_{x}\left(c_{i}\right)$ and $D_{x}\left(c_{i}\right)$ are determined by algebraic equations and thus analytically accessible (see Eqs. 22 and 26–28 in (41)).

The Langevin equations (Eq. 8) possesses a corresponding Fokker-Planck equation that we state here for completeness (for a detailed study of the two-dimensional FPE of IF models with adaptation, see for instance (49,50)):

(9) $$\partial_{t}P\left(c_{i},c_{er},t\right)=LP\left(c_{i},c_{er},t\right)+J_{i}\left(c_{T},c_{er}/\left(1-\varepsilon \right),t\right)\delta \left(c_{i}-c_{R}\right)\text{.}$$

Here, the Fokker-Planck operator is given by

(10) $$L=-\partial_{c_{i}}\left[f\left(c_{i},c_{\text{er}}\right)+D^{\prime }\left(c_{i},c_{\text{er}}\right)-\partial_{c_{i}}D\left(c_{i},c_{\text{er}}\right)\right]-\partial_{c_{\text{er}}}g\left(c_{\text{er}}\right)\text{.}$$

The operator contains the two drift functions $f\left(c_{i},c_{\text{er}}\right)=-\left(c_{i}-c_{i}^{0}c_{\text{er}}\right)/\tau +\mu \left(c_{i},c_{\text{er}}\right)$ and $g\left(c_{\text{er}}\right)=-\left(c_{\text{er}}-1\right)/\tau_{\text{er}}$ as well as the Stratonovich drift $D^{\prime }\left(c_{i},c_{\text{er}}\right)$, where the prime denotes the derivative with respect to $c_{i}$. The IF model’s reset rule in Eq. 8 finds its counterpart in the source term in Eq. 9, which is proportional to the probability current in the $c_{i}$-direction across the threshold:

(11) $$J_{i}\left(c_{T},c_{\text{er}},t\right)=-\partial_{c_{i}}D\left(c_{i},c_{\text{er}}\right)p\left(c_{i},c_{\text{er}},t\right){|}_{c_{i}=c_{T}}\text{.}$$

The factor 1/(1−ε) occurring in Eq. 9 reflects that a trajectory that crosses the threshold at $\left(c_{T},c_{\text{er}}/\left(1-\varepsilon \right)\right)$ is reset to $\left(c_{R},c_{\text{er}}\right)$. In terms of the probability density, this corresponds to a source term at $\left(c_{R},c_{\text{er}}\right)$ proportional to the probability current in the $c_{i}$-direction at $\left(c_{T},c_{\text{er}}/\left(1-\varepsilon \right)\right)$. Finally, Eq. 9 is completed by the absorbing boundary condition

(12) $$P\left(c_{i}=c_{T},c_{\text{er}},t\right)=0\text{,}$$

the natural boundary condition

(13) $$\lim_{c_{i}\to -\infty }P\left(c_{i},c_{\text{er}},t\right)=0\text{,}$$

and the two reflecting boundary conditions

(14) $$J_{\text{er}}\left(c_{i},c_{\text{er}}=0,t\right)=J_{\text{er}}\left(c_{i},c_{\text{er}}=1,t\right)=0\text{,}$$

where $J_{\text{er}}$ is the probability current in the $c_{\text{er}}$-direction. The FPE does in principle allow to calculate statistics as the firing rate r(t) by the probability current across the threshold

(15) $$r\left(t\right)=\int_{0}^{1}dc_{\text{er}}\;J_{i}\left(c_{T},c_{\text{er}},t\right)\text{.}$$

However, the evaluation of this integral is challenging because it requires the solution to Eq. 9, a two-dimensional partial differential equation, which poses a severe problem even numerically (for a discussion of a related problem in the neuroscience context, see (50)).

### Self-consistent firing rate

Instead of attempting to solve the full two-dimensional FPE, we derive approximate expressions for the stationary firing rate $r_{0}$. To this end we assume a static Ca^2+^ concentration in the ER, i.e., we replace $c_{\text{er}}\left(t\right)$ in Eq. 8 by its stationary mean value $\lim_{t\to \infty }\left\langle c_{\text{er}}\left(t\right)\right\rangle =\left\langle c_{\text{er}}^{*}\right\rangle$. In this case, the corresponding FPE is one-dimensional

(16) $$\partial_{t}P\left(c_{i},t\right)=-\partial_{c_{i}}\left[f\left(c_{i}\right)+D^{\prime }\left(c_{i}\right)-\partial_{c_{i}}D\left(c_{i}\right)\right]P\left(c_{i},t\right)+r\left(t\right)\delta \left(c_{i}-c_{R}\right)\text{,}$$

where in all drift and diffusion functions $c_{\text{er}}$ is replaced by $\left\langle c_{\text{er}}^{*}\right\rangle$ (this is omitted for the ease of notation). Solving the stationary one-dimensional FPE is a standard problem in the theory of stochastic processes (51,52) and allows to infer the stationary firing rate $r_{0}$ (see Appendix in Part I):

(17) $$r_{0}\left(\tau ,p,\left\langle c_{\text{er}}^{*}\right\rangle \right)={\left(\int_{c_{R}}^{c_{T}}dc_{1}\;e^{-h\left(c_{1}\right)}\int_{-\infty }^{c_{1}}dc_{2}\;\frac{e^{h\left(c_{2}\right)}}{D\left(c_{2}\right)}\right)}^{-1}$$

with

(18) $$h\left(c\right)=\int_{c_{R}}^{c}dc^{\prime }\;\frac{f\left(c^{\prime }\right)+D^{\prime }\left(c^{\prime }\right)}{D\left(c^{\prime }\right)}\text{.}$$

Solving Eq. 17 requires the knowledge of the stationary mean value $\left\langle c_{\text{er}}^{*}\right\rangle$, which in turn also depends on the firing rate. Thus, Eq. 17 is not sufficient to determine the firing rate $r_{0}$ and we need a second equation that we obtain from a stationary ensemble average of the second line in Eq. 1:

(19) $$0=-\left(\left\langle c_{\text{er}}^{*}\right\rangle -1\right)/\tau_{\text{er}}-\varepsilon \langle c_{\text{er}}^{*}\sum_{i}\delta \left(t-t_{i}\right)\rangle \text{.}$$

This equation involves a conditional mean, $\left\langle c_{\text{er}}^{*}\sum_{i}\delta \left(t-t_{i}\right)\right\rangle =\left\langle c_{\text{er}}^{*}|t=t_{i}^{-}\right\rangle r_{0}$, i.e., the mean ER Ca^2+^ concentration right before the spike multiplied by the firing rate. As shown in the Appendix average of the ER Ca2+ concentration this conditional mean can be approximately related to the unconditional mean by:

(20) $$\left\langle c_{\text{er}}^{*}|t=t_{i}^{-}\right\rangle \approx \left\langle c_{\text{er}}^{*}\right\rangle /\left(1-\varepsilon /2\right)\text{.}$$

**Figure 2:**
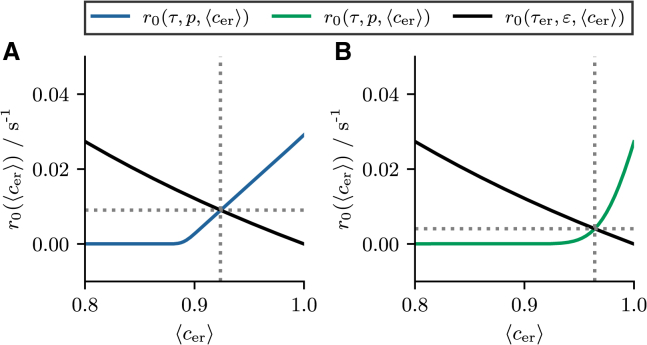
Self-consistent firing rate. (*A* and *B*) Illustrate the self-consistent calculation of the firing rate in the mean-driven and excitable regime, respectively. In each panel, the firing rate $r_{0}\left(\left\langle c_{\text{er}}\right\rangle \right)$ is calculated according to Eq. 17 (*blue* and *green lines*) and Eq. 21 (*black lines*). The intersection of the curves determines the self-consistent firing rate $r_{0}\left(\left\langle c_{\text{er}}^{*}\right\rangle \right)$ and the stationary mean $\left\langle c_{\text{er}}^{*}\right\rangle$, both indicated by dotted lines. Parameters: (*A*) τ=5s, p=0.015, ε=0.03, $\tau_{\text{er}}=300\;s$; (*B*) τ=1s, p=0.06, ε=0.03, $\tau_{\text{er}}=300\;s$. To see this figure in color, go online.

Combining all the relations above, we obtain the desired second relation between the stationary mean value and the firing rate

(21) $$r_{0}\left(\tau_{\text{er}},\varepsilon ,\left\langle c_{\text{er}}^{*}\right\rangle \right)=\frac{1-\left\langle c_{\text{er}}^{*}\right\rangle }{\hat{\varepsilon }\tau_{\text{er}}\left\langle c_{\text{er}}^{*}\right\rangle }\text{,}$$

where $\hat{\varepsilon }=\varepsilon /\left(1-\varepsilon /2\right)$ accounts for the biased sampling problem (53). As illustrated in Fig. 2, the two equations (Eqs. 17 and 21) permit the self-consistent calculation of the firing rate by requiring that both equations are satisfied simultaneously (54). Note that Eq. 17 is a monotonically increasing function of $\left\langle c_{\text{er}}\right\rangle$—the fuller the ER, the higher the firing rate—while Eq. 21 is a monotonically decreasing function of $\left\langle c_{\text{er}}\right\rangle$—the higher the firing rate, the emptier the ER—so that there is only one intersection point and the firing rate is uniquely determined.

**Figure 3:**
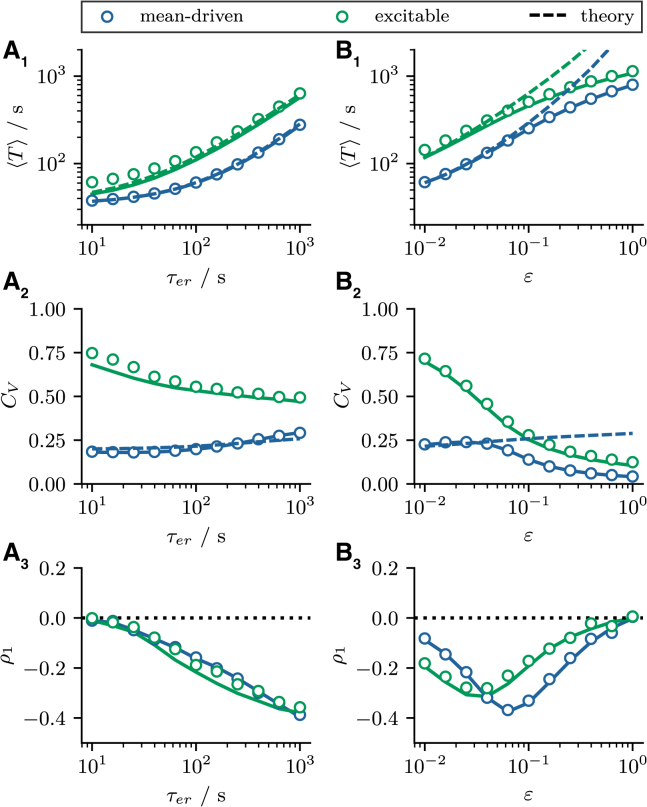
Stationary ISI statistics. (*A* and *B*) Stationary statistics as functions of $\tau_{\text{er}}$ and *ε*, respectively. (*A*_1_ and *B*_1_) The mean ISI ⟨T⟩. (*A*_2_ and *B*_2_) The coefficient of variation $C_{V}$. (*A*_3_ and *B*_3_) The serial correlation coefficient $\rho_{1}$. Blue/green circles and solid lines indicate statistics calculated from stochastic simulations of the two-component model (Eq. 1) and the Langevin approximation (Eq. 8), respectively. Blue/green dashed lines indicate theoretical predictions according to Eqs. 17, 21, and 22. Parameters: (mean driven) τ=5s, p=0.015; (excitable) τ=1s, p=0.06; (*A*) ε=0.03; (*B*) $\tau_{\text{er}}=300\;s$. To see this figure in color, go online.

In Fig. 3, *A*_1_ and *B*_1_ we compare the mean ISI obtained from stochastic simulations of the two-component model (*circles*) and the Langevin model (*solid lines*) to the theoretical prediction of the mean ISI (inverse firing rate) $\left\langle T\right\rangle =1/r_{0}\left(\left\langle c_{\text{er}}^{*}\right\rangle \right)$ calculated from Eqs. 17 and 21 (*dashed lines*). First of all, we note that the Langevin approximation provides a good description of the full model in both the mean-driven (*blue circles* and *lines*) and excitable (*green circles* and *lines*) firing regime for all parameters. Moreover, we find excellent agreement between simulation results and theory for all values of $\tau_{\text{er}}$ and for small values of *ε* (*solid* and *dashed lines* are almost indistinguishable). The fact that *ε* rather than $\tau_{\text{er}}$ is the limiting factor may be surprising at first, since the self-consistent method relies on the assumption that $c_{\text{er}}\left(t\right)$ changes slowly—a property that one could intuitively ascribe to the parameter $\tau_{\text{er}}$. However, in the stationary state, the amplitude of the depletion, determined by the parameter *ε*, and the replenishment, determined by the parameter $\tau_{\text{er}}$, must balance on average over an ISI. Therefore, the parameter *ε* does not only specify how strongly $c_{\text{er}}\left(t\right)$ is decreased when a spike is fired, but also how much $c_{\text{er}}\left(t\right)$ increases again over an ISI. Therefore, *ε* has to be small so that it can be assumed that $c_{\text{er}}\left(t\right)$ is constant over an ISI.

The mean-adaptation approximation does in principle also allow to calculate the variance $\left\langle \delta T^{2}\right\rangle$ of the ISI:

(22) $$\left\langle \delta T^{2}\right\rangle =2\int_{-\infty }^{c_{T}}dx_{3}\;e^{-h\left(x_{3}\right)}{\left(\int_{-\infty }^{x_{3}}dx_{2}\;\frac{e^{h\left(x_{2}\right)}}{D\left(x_{2}\right)}\right)}^{2}\times \int_{x_{3}}^{c_{T}}dx_{1}\;e^{-h\left(x_{1}\right)}\Theta \left(x_{1}-c_{R}\right)\text{,}$$

and thus also the CV $C_{V}=\left\langle T\right\rangle /\sqrt{\left\langle \delta T^{2}\right\rangle }$. However, the time dependence of $c_{\text{er}}\left(t\right)$ is often crucial for the variability of the ISI and cannot be neglected. As a result, the mean-adaptation approximation captures the CV only in special cases (see Fig. 3, *A*_2_ and *B*_2_).

### Coefficient of variation

A remarkable feature of Ca^2+^ signaling is the persistence of the CV across different cells of the same cell line stimulated by a certain agonist, despite considerable cell-to-cell variability (4,12). This experimental finding was difficult to reproduce with our previous nonadaptive model ($c_{\text{er}}\left(t\right)=\text{const.}$), where the ISI variability stems solely from the fluctuation of the cytosolic Ca^2+^ concentration, that is caused by the stochasticity of the puff current $j_{\text{puff}}\left(c_{i},c_{\text{er}}\right)$, resulting from the stochastic opening and closing of clusters of IP_3_ receptor channels. As a result, the noise acting on the cytosolic Ca^2+^ concentration was relatively weak and spiking is either nearly deterministic in the mean-driven regime or highly stochastic in the excitable regime. Introducing an adaptation variable solves this problem to some extent because it introduces a second source of ISI variability by varying the initial conditions $c_{i}\left(t_{i}^{+}\right)$ and $c_{\text{er}}\left(t_{i}^{+}\right)$, potentially increasing the CV, but it also introduces a relative refractory period, potentially decreasing the CV. Which of these effects dominates the ISI statistics depends on the specific choice of parameters.

In Fig. 3, *A*_2_ and *B*_2_ we plot the CV as a function of the parameters $\tau_{\text{er}}$ and *ε*, respectively. For small values of $\tau_{\text{er}}$ and *ε*, the model behaves very similarly to the one without adaptation. In this case, the CV is approximately $C_{V}\approx 0.25$ for the specific parameters in the mean-driven regime (*blue circles*) and $C_{V}\approx 0.75$ for the specific parameters in the excitable regime (*green circles*). In the mean-driven regime, we find that the CV depends nonmonotonically on the two parameters. As $\tau_{\text{er}}$ is increased, the CV decreases very slightly at first, before the CV increases as $\tau_{\text{er}}$ is increased further. Increasing the parameter *ε* has the opposite effect, the CV first increases very slightly before decreasing as *ε* is increased further. In the excitable regime, we observe a general decrease of the CV with increasing strength of the adaptation, regardless of whether this is achieved by varying $\tau_{\text{er}}$ or *ε*. In any case, the introduction of an additional adaptive variable results in a less-pronounced difference in the ISI variability between the mean-driven and excitable regimes compared with the nonadaptive model.

**Figure 4:**
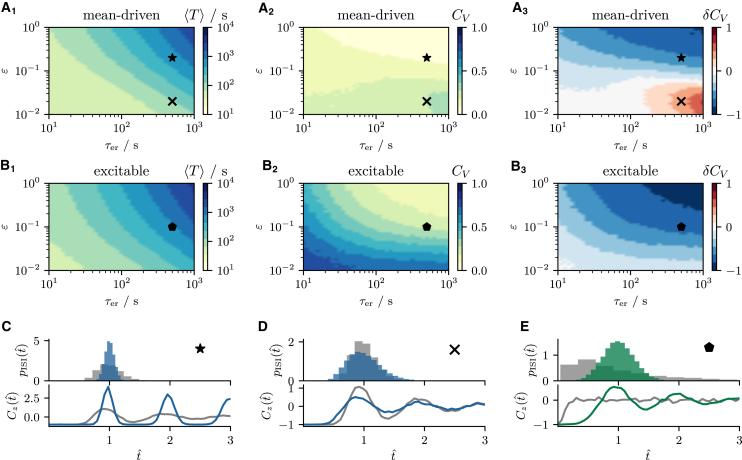
Coefficient of variation in the presence of an adaptation variable. (*A* and *B*) The mean ISI ⟨T⟩, CV $C_{V}$, and the relative change of the CV compared with the nonadaptive case $\delta C_{V}=\left(C_{V}-C_{V}^{*}\right)/C_{V}^{*}$. $C_{V}^{*}$ denotes the CV of the nonadaptive model with similar parameters but ε=0. Increasing $\tau_{\text{er}}$ or *ε* leads to a prolongation of the mean ISI. The effect on the CV depends on the choice of parameters in the mean-driven regime (*A*_2_ and *A*_3_). It is decreased for $\tau_{\text{er}}=500\;s$ and ε=0.2 (⋆) and increased for τ=500s and ε=0.02 (×). The CV is generally decreased in the excitable regime (*B*_2_ and *B*_3_) as illustrated for τ=500s and ε=0.1 (). (*C*–*E*) The ISI densities $p_{\text{ISI}}\left(\hat{t}\right)$ and spike train correlation $C_{z}\left(\hat{t}\right)$ for the nonadaptive (gray histograms and lines) and the adaptive model (*blue/green histograms* and *lines*) as functions of the rescaled time $\hat{t}=t/\left\langle T\right\rangle$ corresponding to the three cases. Parameters: (mean-driven) τ=5s, p=0.015; (excitable) τ=1s, p=0.06; and (*C*) $\tau_{\text{er}}=500\;s$, ε=0.2; (*D*) $\tau_{\text{er}}=500\;s$, ε=0.02; (*E*) $\tau_{\text{er}}=500\;s$, ε=0.1. To see this figure in color, go online.

To explain why this is the case, we show in Fig. 4, *A* and *B* the mean ⟨T⟩, CV $C_{V}$, and the relative change of the CV compared with the nonadaptive case $\delta C_{V}=\left(C_{V}-C_{V}^{*}\right)/C_{V}^{*}$ as functions of $\tau_{\text{er}}$ and *ε*; here $C_{V}^{*}$ denotes the CV without adaptation (ε=0) but with the remaining parameters unchanged.

In the mean-driven regime for a significant depletion during a single spike (ε>0.1), the CV decreases compared with the nonadaptive case irrespective of the choice of $\tau_{\text{er}}$ (see Fig. 4 *A*_3_). This is so because larger values of *ε* result in a strong inhibition right after a spike, causing an effective increase in the refractory period and thus a more regular spike train (55). In Fig. 4 *C* we compare the ISI density $p_{\text{ISI}}\left(t\right)$ and the spike train autocorrelation function $C_{z}\left(t\right)=\left\langle z\left(t^{\prime }+t\right)z\left(t^{\prime }\right)\right\rangle -{\left\langle z\left(t^{\prime }\right)\right\rangle }^{2}$ between the nonadaptive (*gray histogram* and *line*) and the adaptive model (*blue histogram* and *line*) with adaptation parameters as highlighted by the star in Fig. 4 *A*. Because the depletion of the ER strongly affects the mean ISI, we normalize the time axis by ⟨T⟩ in the adaptive and nonadaptive cases. Consistent with our reasoning, the ISI density and the correlation function are more strongly peaked for the adaptive model.

When the depletion during a single spike is small (ε<0.1) and the replenishment is slow, the CV can even be increased in the mean-driven regime (e.g., for parameters highlighted by the *cross* in Fig. 4 *A*_3_). The corresponding ISI density and correlation function are broadened (see Fig. 4 *D*). Here, $c_{\text{er}}\left(t\right)$ varies only weakly around its mean value $\left\langle c_{\text{er}}^{*}\right\rangle$. Because this mean value is smaller than the initial value $c_{\text{er}}\left(t\right)=1$ the system is effectively poised closer to the bifurcation than without adaptation, which accounts for the increase of the CV in this parameter regime.

Finally, in the excitable regime we observe a general reduction of the CV for all combinations of $\tau_{\text{er}}$ and *ε*, most likely due to an increase in the effective refractory period, as mentioned above. The additional refractory period will always reduce the CV compared with the nonadaptive case, where spiking is almost Poissonian. The increase in the temporal precision is illustrated in Fig. 4 *E* by means of the ISI density and correlation function of the adaptive model. Both statistics show characteristics of a more regular spiking process, often associated with the mean-driven regime but here mediated by the adaptation variable.

### Second-order stationary statistics

So far, we have mainly considered the mean and CV, two first-order stationary ISI statistics. When the intervals are statistically independent and identically distributed, i.e., when we are dealing with a renewal point process, the ISI sequence is completely characterized by the probability density (56,57). However, a common feature of models with spike-frequency adaptation is that the ISIs are *not* statistically independent, but correlated (58,59,60,61,62)—the adaptation variable, here $c_{\text{er}}\left(t\right)$, keeps a memory beyond the single ISI.

**Figure 5:**
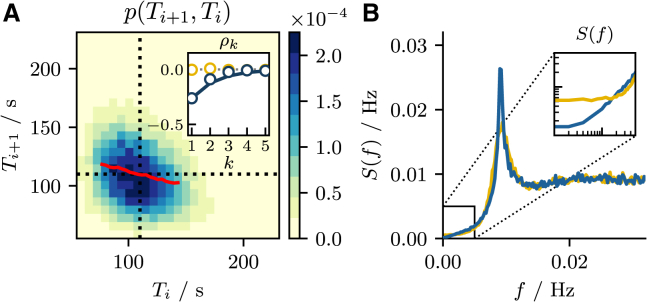
Second-order stationary ISI statistics. (*A*) The joint probability density function $p\left(T_{i+1},T_{i}\right)$ together with the conditional mean $\left\langle T_{i+1}|T_{i}\right\rangle$ (*red line*). They demonstrate an anticorrelation between adjacent intervals as confirmed by $\rho_{1}<0$ in the inset. The inset shows the serial correlation coefficient (SCC) $\rho_{k}$ for the ordered ISI sequence *(blue circles*) and for the shuffled ISI sequence (*yellow circles*). (*B*) The power spectrum S(f) again for the ordered (*blue line*) and shuffled ISI sequence (*yellow line*). The inset in (*B*) shows a zoom-in on the low-frequency region, where the spectrum of the ordered sequence has reduced power due to negative interval correlations (Eq. 27). Parameters as in Fig. 1. To see this figure in color, go online.

The ISIs in our model are indeed correlated. The full picture of the interdependence between subsequent intervals is given by the joint probability density $p\left(T_{i+1},T_{i}\right)$ shown in Fig. 5 *A*. However, the anticorrelation between adjacent intervals is more clearly seen by the conditional mean $\left\langle T_{i+1}|T_{i}\right\rangle$ (*red line*). Specifically, the longer (shorter) the interval $T_{i}$ is, the shorter (longer) is the subsequent interval $T_{i+1}$ on average.

As we have briefly mentioned in the introduction, interval correlations over several lags *k* can be quantified by the SCC

(23) $$\rho_{k}=\frac{\left\langle \delta T_{i+k}\delta T_{i}\right\rangle }{\left\langle \delta T_{i}^{2}\right\rangle }$$

where $\delta T_{i}=T_{i}-\left\langle T\right\rangle$ is the deviation of the *i*-th ISI $T_{i}$ from the mean. If the SCC is positive, ($\rho_{k}>0$) intervals are said to be (positively) correlated. This is the case if, on average, two intervals $T_{i}$, $T_{i+k}$ deviate similarly from the mean, i.e., if both intervals are longer (or shorter) than the mean, so that the sign of the respective deviations $\delta T_{i}$, $\delta T_{i+k}$ agree. On the contrary, if the SCC is negative ($\rho_{k}<0$) intervals are said to be anticorrelated and a long interval is usually followed by a short interval (or vice versa). Finally, if the SCC vanishes ($\rho_{k}=0$) the intervals are uncorrelated; for a discussion of the different cases, see (63). The first SCC $\rho_{1}$ as a function of $\tau_{\text{er}}$ and *ε* is shown in Fig. 3, *A*_3_ and *B*_3_, respectively. We note that this coefficient is always negative, as expected in the presence of a spike-triggered adaptation (58,59,60,61,64,65). Furthermore, $\rho_{1}$ decreases monotonically as a function of $\tau_{\text{er}}$ and exhibits a local minimum as a function of *ε*. To understand the latter feature, we point out that correlations must be absent in both limit cases ε=0 (no adaptation) and ε=1 (complete depletion) and we can expect to find a maximal strength of correlation for intermediate values of *ε*. The fact that intervals are uncorrelated for ε=1 is because in this case the ER is always completely depleted, regardless of the length of the intervals. Going beyond the first SCC, in the inset of Fig. 5 *A* we show the sequence of correlation coefficients $\rho_{k}$ over the lag *k* (*blue circles*) and observe an anticorrelation that decays monotonically over a few lags according to

(24) $$\rho_{k}\approx \rho_{1}e^{-\left(k-1\right)/n_{\text{corr}}}$$

where $n_{\text{corr}}$ is the number of correlated intervals. The fact that for our model the SCC monotonically approaches 0 as *k* increases, can be well understood in the mean-driven regime. Schwalger and Lindner (61) have shown that in this regime the pattern of interval correlations is related to the mean drift $f\left(c_{i},c_{\text{er}}\right)$ at the reset point: for a positive drift the correlations do not change sign with the lag *k*. For our model, the drift at the reset point is given by the mean puff current $f\left(c_{R},c_{\text{er}}\right)=\mu \left(c_{R},c_{\text{er}}\right)>0$, which is always positive.

The monotonic decay of $\rho_{k}$ has consequences for the first SCC $\rho_{1}$ because the sum over all SCCs is bound according to (see below for an explanation)

(25) $$\sum_{k=1}^{\infty }\rho_{k}\geq -1/2\text{.}$$

This implies $\rho_{1}>-1/2$ when all SCCs are negative. Indeed in Fig. 3, *A*_3_ and *B*_3_ we do not observe an SCC $\rho_{1}$ smaller than −1/2, neither in the mean-driven nor in the excitable regime. Combining Eqs. 24 and 25, evaluating the sum and rearranging terms, allows to derive an upper limit for the number of correlated intervals:

(26) $$n_{\text{corr}}\geq -\frac{1}{\ln\left(1+2\rho_{1}\right)}\text{,}$$

given $-1/2\leq \rho_{1}\leq 0$. This implies that, if adjacent intervals are strongly anticorrelated, the number of correlated intervals $n_{\text{corr}}$ is small, and leads to the somewhat counterintuitive conclusion that, because $\left|\rho_{1}\right|$ increases with $\tau_{\text{er}}$ (Fig. 3 *A*_3_), the number of correlated intervals $n_{\text{corr}}$ decreases asymptotically as $\tau_{\text{er}}\to \infty$ (not shown).

While the SCC is an interesting statistic on its own, it is also important because it shapes spectral measures and therefore has consequences for information transmission and signal detection (63,64,66,67,68,69,70). Specifically, the low-frequency limit of the spike train power spectrum is given by (56):

(27) $$\lim_{f\to 0}S\left(f\right)=r_{0}C_{V}^{2}\left(1+2\sum_{k=1}^{\infty }\rho_{k}\right)$$

with $S\left(f\right)=\lim_{T\to \infty }\left\langle {\left|\tilde{z}\left(f\right)\right|}^{2}\right\rangle /T$ and the Fourier transform of the spike train $\tilde{z}\left(f\right)=\int_{0}^{T}dtz\left(t\right)\;\exp\;\left(2\pi ift\right)$. The factor in parentheses leads to a reduction if the sum over $\rho_{k}$ is negative. Because the power spectrum has to be positive, we can conclude from Eq. 27 that Eq. 25 must hold. A reduced power at low frequencies due to negative interval correlations can thus improve the signal/noise ratio in the presence of a slow signal (67,68). This reduction is demonstrated in Fig. 5 *B* where we compare the power spectrum S(f) of the original spike train (*blue*) to the power spectrum of the same spike train with all ISIs randomly shuffled (*orange*). Shuffling the sequence of ISIs provides a simple method to decorrelate intervals without changing first-order statistics (see inset Fig. 5 *A*). As a consequence, the power spectrum of the shuffled spike train has larger power at low frequencies compared with the original spike train (see inset Fig. 5 *B*).

#### Timescale of the transient: Ca^2+^ depletion

The process that gives rise to the time dependence of the spiking statistics is the cumulative depletion of the ER Ca^2+^ concentration over several spikes. It is therefore suggestive to consider the timescale on which the variable $c_{\text{er}}\left(t\right)$ approaches its stationary value to estimate the length of the transient. This effective timescale $\tau_{\text{eff}}$ at which the ER is depleted is *not* to be confused with the timescale $\tau_{\text{er}}$ at which the ER is replenished in the absence of spikes.

**Figure 6:**
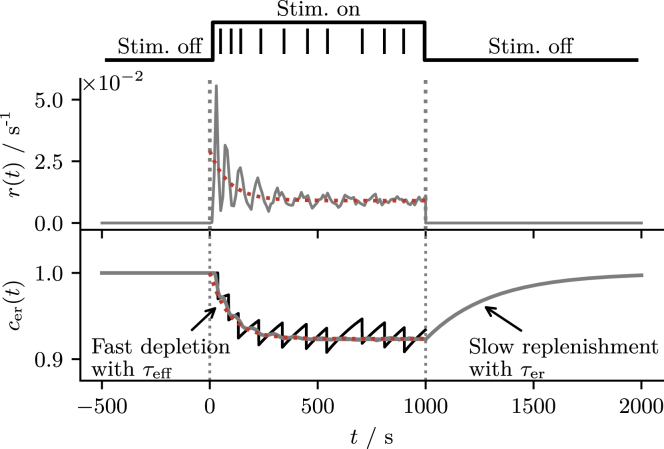
Timescales of ER depletion and replenishment. Response of the firing rate r(t) and ER Ca^2+^ concentration $c_{\text{er}}\left(t\right)$ to a constant IP_3_ stimulation presented over a period of time. The IP_3_ stimulation is indicated by the upper black step function—turned on at t=0s and turned off at t=1000s. Spike times of a single realization and the corresponding time series $c_{\text{er}}\left(t\right)$ are indicated by black lines. The firing rate r(t) and ensemble average $\left\langle c_{\text{er}}\left(t\right)\right\rangle$ are shown by gray lines. The effective timescale $\tau_{\text{eff}}$ at which $\left\langle c_{\text{er}}\left(t\right)\right\rangle$ approaches its stationary value in response to the onset of the stimulus does not match the timescale $\tau_{\text{er}}$ at which $\left\langle c_{\text{er}}\left(t\right)\right\rangle$ approaches its stationary value in the absence of the stimulus. Dotted red lines show the theory where $\left\langle c_{\text{er}}\left(t\right)\right\rangle$ is calculated according to Eq. 31 and $r_{0}\left(\left\langle c_{\text{er}}\left(t\right)\right\rangle \right)$ according to Eq. 17. Parameters as in Fig. 1. To see this figure in color, go online.

We apply a constant IP_3_ stimulation for a certain time (*top black line*) to the model in Fig. 6. That evokes Ca^2+^ spikes. The sequence of spike times is indicated by vertical black lines. The corresponding time series of the variable $c_{\text{er}}\left(t\right)$ is shown in the lower panel by a black line. Apparently, the reduction of $c_{\text{er}}\left(t\right)$ with each spike significantly lengthens the ISIs.

We estimate $\tau_{\text{eff}}$ on the basis of the time-dependent ensemble average $\left\langle c_{\text{er}}\left(t\right)\right\rangle$ obtained from a large number $N_{\text{sim}}$ of numerical simulations. All of them started at $c_{\text{er},n}\left(0\right)=1$, $c_{i,n}\left(0\right)=c_{R}$ and with all clusters in the state $0_{1}$. We estimate $\left\langle c_{\text{er}}\left(t\right)\right\rangle$ from the individual time courses $c_{\text{er},n}\left(t\right)$ by $\left\langle c_{\text{er}}\left(t\right)\right\rangle \approx \sum_{n=1}^{N_{\text{sim}}}c_{\text{er},n}\left(t\right)/N_{\text{sim}}$. Similarly, the instantaneous firing rate r(t) can be estimated by the fraction of realizations $c_{i,n}\left(t\right)$ that cross the firing threshold $c_{T}$ in a small time bin [t,t+Δt] divided by Δt. The firing rate r(t) and the average $\left\langle c_{\text{er}}\left(t\right)\right\rangle$ are shown in Fig. 6 by gray lines. While the rate r(t) is subject to a significant ringing, the time-dependent adaptation variable $\left\langle c_{\text{er}}\left(t\right)\right\rangle$ is a rather smooth function of time.

**Figure 7:**
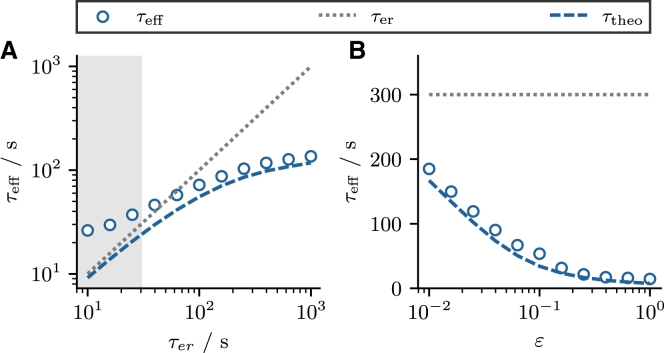
Effective timescale of ER depletion. (*A* and *B*) The effective timescale $\tau_{\text{eff}}$ measured from numerical simulations (*blue circles*) compared with $\tau_{\text{er}}$ (*gray dotted line*) the first-order estimate $\tau_{\text{theo}}$ (*dashed red line*). The gray area in (*A*) indicates the region where the mean initial ISI $\left\langle T_{0}\right\rangle$ falls below the timescale $\tau_{\text{er}}$. In this case, the measured timescale reflects the mean ISI rather than the actual effective timescale. Parameters as in Fig. 1. To see this figure in color, go online.

To define the effective timescale $\tau_{\text{eff}}$, we assume that the time course of $\left\langle c_{\text{er}}\left(t\right)\right\rangle$ can be fit by a single exponential function

(28) $$\left\langle c_{\text{er}}^{*}\right\rangle +\left(1-\left\langle c_{\text{er}}^{*}\right\rangle \right)e^{-t/\tau_{\text{eff}}}\text{,}$$

from which we obtain $\tau_{\text{eff}}$ as a fit parameter. This estimate of $\tau_{\text{eff}}$ is shown in Fig. 7 (*blue circles*) versus $\tau_{\text{er}}$ and *ε*, dependencies which are discussed below. Here, we just point out that generally, $\tau_{\text{eff}}$ differs significantly from $\tau_{\text{er}}$.

To obtain an analytical estimate of the effective timescale, we consider the dynamics of the time-dependent mean $\left\langle c_{\text{er}}\left(t\right)\right\rangle$. To this end, we take the ensemble average of the second line in Eq. 1, which leads to the approximate relation:

(29) $$\frac{d\left\langle c_{\text{er}}\right\rangle }{dt}\approx -\left(\left\langle c_{\text{er}}\right\rangle -1\right)/\tau_{\text{er}}-\hat{\varepsilon }\left\langle c_{\text{er}}\right\rangle r_{0}\left(\left\langle c_{\text{er}}\right\rangle \right)\text{.}$$

Here, we have made two approximations. First, we have assumed that the relation between the unconditional and conditional mean given by Eq. 20 does hold for all times. Second, we have substituted the time-dependent firing rate by the stationary rate taken at the instantaneous value of $\left\langle c_{\text{er}}\left(t\right)\right\rangle$, i.e., we use $r\left(t\right)\approx r_{0}\left(\left\langle c_{\text{er}}\left(t\right)\right\rangle \right)$. Despite these simplifications, Eq. 29 cannot be solved analytically because the functional dependence of the stationary firing rate on $\left\langle c_{\text{er}}\right\rangle$, although in principle known from Eq. 17, is complicated. To find an approximate solution for $\left\langle c_{\text{er}}\left(t\right)\right\rangle$, we expand the firing rate around $\left\langle c_{\text{er}}^{*}\right\rangle$ in first order, $r_{0}\left(x\right)\approx r_{0}\left(x^{*}\right)+r_{0}^{\prime }\left(x^{*}\right)\left(x-x^{*}\right)$. Since the expressions derived below become somewhat lengthy, we introduce here the abbreviation $x\left(t\right)=\left\langle c_{\text{er}}\left(t\right)\right\rangle$ (and $x^{*}=\left\langle c_{\text{er}}^{*}\right\rangle$). By expanding the rate up to the first order in x(t), Eq. 29 becomes a quadratic differential equation:

(30) $$\dot{x}=-\left(x-1\right)/\tau_{\text{er}}-\hat{\varepsilon }x\left[r_{0}\left(x^{*}\right)+r_{0}^{\prime }\left(x^{*}\right)\left(x-x^{*}\right)\right]$$

that is solved by:

(31) $$x\left(t\right)=x^{*}\frac{\left(x_{0}+x^{*}\right)+\left(x_{0}-x^{*}\right)e^{-t/\tau_{\text{theo}}}}{\left(x_{0}+x^{*}\right)-\left(x_{0}-x^{*}\right)e^{-t/\tau_{\text{theo}}}}$$

with the initial condition $x_{0}=1$ and timescale

(32) $$\tau_{\text{theo}}=\frac{\tau_{\text{er}}}{\sqrt{{\left(1+\hat{\varepsilon }\tau_{\text{er}}\left[r_{0}\left(x^{*}\right)-r_{0}^{\prime }\left(x^{*}\right)x^{*}\right]\right)}^{2}+4\hat{\varepsilon }\tau_{\text{er}}r_{0}^{\prime }\left(x^{*}\right)}}\text{.}$$

We note that, strictly speaking, one cannot expect that the two timescales $\tau_{\text{theo}}$ and $\tau_{\text{eff}}$ coincide because the functions used to calculate and measure them are different. However, Eq. 31 can be expressed in terms of $\Delta x=x_{0}-x^{*}$ and—similar to the firing rate—expanded up to the first order in Δx to obtain a single exponential again

(33) $$x\left(t\right)\approx x^{*}+\left(x_{0}-x^{*}\right)e^{-t/\tau_{\text{theo}}}\text{.}$$

In other words if the difference $1-\left\langle c_{\text{er}}^{*}\right\rangle$ is small, i.e., if the cumulative depletion of the ER is not too strong, we can approximate $\tau_{\text{eff}}$ by $\tau_{\text{theo}}$.

In Fig. 7 we compare the effective timescale $\tau_{\text{eff}}$ with $\tau_{\text{theo}}$ as functions of $\tau_{\text{er}}$ and *ε*. We find good agreement over a broad range of parameters, except for small values of $\tau_{\text{er}}$ (*gray area* in Fig. 7 *A*). This is not so much a failure of the theory as it is a failure of the estimation of $\tau_{\text{eff}}$ from the simulation data. When the effective timescale becomes as short as the first ISI, synchronization-induced ringing effects in the firing rate become also noticeable in the response of $\left\langle c_{\text{er}}\left(t\right)\right\rangle$ and lead to the observed deviations. Regarding the shown functional dependence it is remarkable that the effective timescale drops monotonically with *ε* and that this dependence is much stronger than the one on $\tau_{\text{er}}$.

Finally, we note that in Fig. 7 we have compared $\tau_{\text{eff}}$ with $\tau_{\text{theo}}$ when the model operates in the mean-driven regime. Here, we find that $\tau_{\text{theo}}$ provides a good approximation to the effective timescale. This has to do with the fact that, in the mean-driven regime $r_{0}\left(\left\langle c_{\text{er}}\right\rangle \right)$ can be well approximated by a linear function (cf. Fig. 2 *A*). This is not the case in the excitable regime (cf. Fig. 2 *B*), so that there the estimate becomes worse (not shown).

### Transient interspike interval statistic

The previously developed methods for the estimation of the effective timescale $\tau_{\text{eff}}$ are based on the firing rate r(t) obtained from a large ensemble. This is not always experimentally feasible. We thus return to a description of the ISI, this time during the transient. Already in the first section, we have mentioned that the sequence of mean ISIs $\left\{\left\langle T_{i}\right\rangle \right\}$ can be well fit by a single exponential function Eq. 7. Here, we provide an estimate for the two parameters, the cumulative refractory period ΔT and the number of transient intervals $n_{\text{tr}}$, based on the stationary firing rate and the effective timescale of ER depletion.

The calculation of the first ISI $T_{0}=\left\langle T_{0}\right\rangle$ and the stationary ISI $T_{\infty }=\left\langle T\right\rangle$ is straightforward. Initially, the ER is completely filled so that we can calculate the initial mean interval using Eq. 17 with $\left\langle c_{\text{er}}\right\rangle =1$:

(34) $$T_{0}=1/r_{0}\left(\left\langle c_{\text{er}}\right\rangle =1\right)\text{.}$$

The stationary interval $T_{\infty }$ is given by the inverse of the stationary rate (Eqs. 17 and 21):

(35) $$T_{\infty }=1/r_{0}\left(\left\langle c_{\text{er}}\right\rangle =\left\langle c_{\text{er}}^{*}\right\rangle \right)\text{.}$$

**Figure 8:**
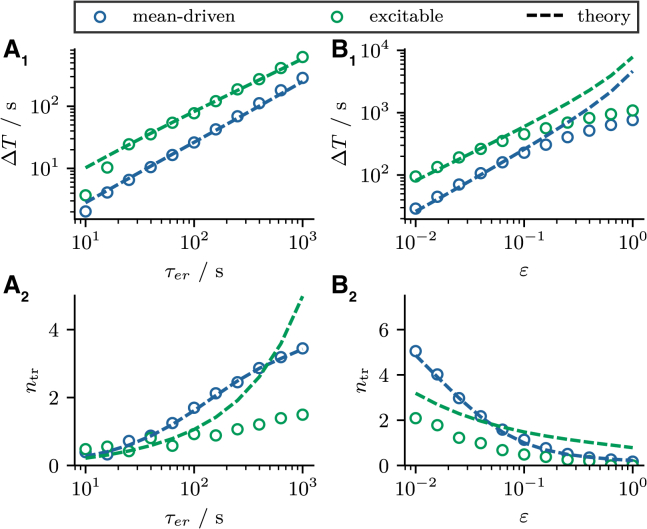
Transient ISI statistics. (*A* and *B*) Transient statistics as functions of $\tau_{\text{er}}$ and *ε*, respectively. (*A*_1_ and *B*_1_) The cumulative refractory period ΔT. (*A*_2_ and *B*_2_) The number of transient intervals $n_{\text{tr}}$. Blue and green circles indicate statistics calculated from stochastic simulations of the two-component model (Eq. 1). Blue and green lines indicate theoretical predictions according to Eqs. 34, 35, and 36. Parameters: (mean-driven) τ=5s, p=0.015; (excitable) τ=1s, p=0.06; (*A*) ε=0.03; (*B*) $\tau_{\text{er}}=300\;s$. To see this figure in color, go online.

In Fig. 8, *A*_1_ and *B*_1_ we compare the theoretical prediction for $\Delta T=T_{\infty }-T_{0}$ according to Eqs. 34 and 35 (*blue* and *green lines*) to the cumulative refractory period obtained by fitting the sequence of mean intervals $\left\{\left\langle T_{i}\right\rangle \right\}$ by Eq. 7 (*blue* and *green circles* for mean-driven and excitable regime, respectively). We find good agreement between simulation results and theory over a broad range of parameter values except for large *ε* (this deviation was explained in the context of the stationary mean interval). The cumulative refractory period increases both with $\tau_{\text{er}}$ and *ε*, reflecting the increase of the stationary interval ($T_{0}$ is independent of $\tau_{\text{er}}$ and *ε*).

We use the approximation of the effective timescale $\tau_{\text{eff}}\approx \tau_{\text{theo}}$ derived in the previous section to estimate the number of transient intervals $n_{\text{tr}}$

(36) $$n_{\text{tr}}\approx \tau_{\text{eff}}/T_{0}\text{.}$$

This ratio indicates how many nonadaptive intervals $T_{0}$ the transient contains, providing an upper bound on $n_{\text{tr}}$ because $\left\langle T_{0}\right\rangle \leq \left\langle T_{i}\right\rangle$. In Fig. 8, *A*_2_ and *B*_2_ we show the number of transient intervals $n_{\text{tr}}$ obtained from the aforementioned fit procedure (*blue* and *green circles*) and compare it with the estimate Eq. 36 (*blue* and *green lines*). We find good agreement if the model operates in the mean-driven regime (*blue circles*) but some disagreement in the excitable regime (*green circles*). The deviations in the excitable case are not too surprising because the approximation of $\tau_{\text{eff}}\approx \tau_{\text{theo}}$ is based on the linearization of the stationary firing rate $r_{0}\left(\left\langle c_{\text{er}}\right\rangle \right)$, which is convincing in the mean-driven but not in the excitable regime (see Fig. 2). Note that the number of transient intervals increases only moderately with $\tau_{\text{er}}$. For the chosen parameters, even for large values of the timescales $\tau_{\text{er}}\approx 10^{3}$, only a relatively small number of transient intervals $n_{\text{tr}}\approx 3$ is observed. Somewhat surprisingly, larger numbers of transient intervals can be realized when the parameter *ε* is decreased.

**Figure 9:**
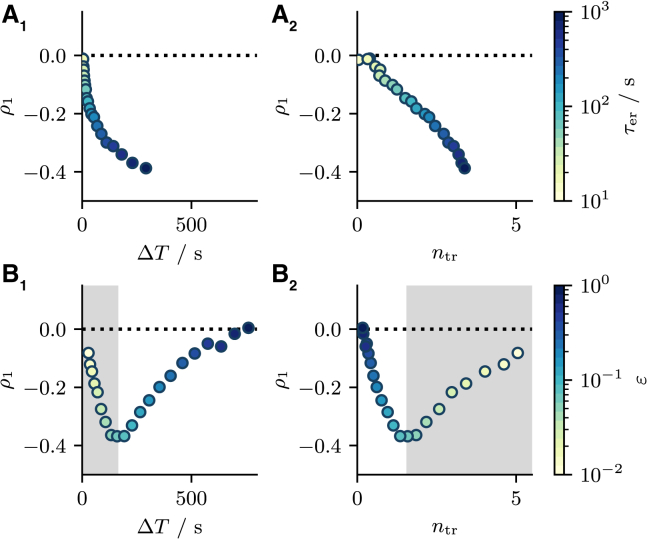
Stationary over transient ISI statistics. (*A*_1_ and *A*_2_) The SCC $\rho_{1}$ as a function of the cumulative refractory period ΔT and the number of transient intervals $n_{\text{tr}}$ when the parameter $\tau_{\text{er}}$ is varied. In both cases, interval correlations depend monotonically on both $n_{\text{tr}}$ and ΔT. (*B*_1_ and *B*_2_) $\rho_{1}$ as a function of ΔT and $n_{\text{tr}}$ when *ε* is varied. Here, $\rho_{1}$ is a nonmonotonic function of $n_{\text{tr}}$ and ΔT due to the nonmonotonic dependence of the transient statistics on *ε*. The gray area indicates small values of ε<0.1. Parameters: τ=5s, p=0.015, and (*A*) ε=0.03, (*B*) $\tau_{\text{er}}=300\;s$. To see this figure in color, go online.

We also examine how the transient statistics are connected to the SCC of the stationary intervals. The relations between the SCC $\rho_{1}$ on the one hand and the cumulative refractory period ΔT or the number of transient intervals $n_{\text{tr}}$ on the other hand are simple if varied through $\tau_{\text{er}}$: the more pronounced the transient the stronger the stationary ISI correlations (see Fig. 9, *A*_1_ and *A*_2_). The relations become more complicated if *ε* is varied (see Fig. 9, *B*_1_ and *B*_2_) because the SCC exhibits a minimum versus *ε* (cf. Fig. 3 *B*_3_). Consequently, $\rho_{1}$ displays a minimum versus ΔT and $n_{\text{tr}}$, and we can subdivide the $\rho_{1}$ curve into a small *ε* region (*gray area* in Fig. 9, *B*_1_ and *B*_2_) and a large *ε* region (*white area* in the same panels). Specifically, the gray area in Fig. 9 *B*_2_ illustrates that for small *ε* an increase in the number of transient intervals comes along with diminished interval correlations $\rho_{1}$. Last but not least, another feature for small *ε* is that interval correlations increase with growing cumulative refractory period ΔT irrespective of whether *ε* or $\tau_{\text{er}}$ are varied (see Fig. 9, *A*_1_ and *B*_1_). We will return to these observations in the next section.

**Figure 10:**
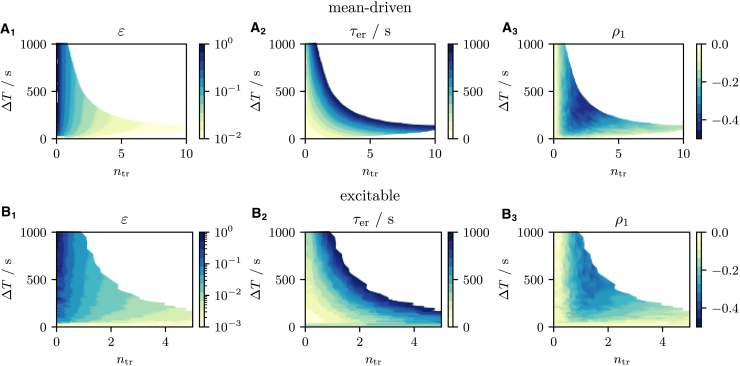
Summary of the behavior of the slow store Ca^2+^ dynamics. (*A* and *B*) The relations between the system parameters *ε* varied from 10^−3^ to 1 and $\tau_{\text{er}}$ varied from 0s to 1000s and the behavioral characteristics $n_{\text{tr}}$, ΔT, and $\rho_{1}$ in the mean-driven (*A*) and excitable (*B*) regime. White regions in the plots show values of ($n_{\text{tr}}$, ΔT) pairs which cannot be realized with $\tau_{\text{er}}\leq 1000\;s$. Increasing $\tau_{\text{er}}$ beyond 1000s has little effect on the boundaries of these regions. Long transients (large $n_{\text{tr}}$) often indicate a small net loss (small *ε*) and slow replenishment (large $\tau_{\text{er}}$). Large cumulative refractory periods ΔT can be realized by a large net loss or slow replenishment. Interval correlation coefficients $\rho_{1}$ are maximized for an intermediate number of transient intervals and intermediate cumulative refractory periods. To see this figure in color, go online.

Finally, we ask what conclusions can be drawn about the adaptation parameters when the statistics of the transient are known. In principle, this also depends on the two model parameters *τ* and *p*, which determine whether the model is mean driven or excitable, but we find that the results are qualitatively similar in the two firing regimes. Generally, long transients (large $n_{\text{tr}}$) coincide with a small net loss of Ca^2+^ (small *ε*) and a slow replenishment (large $\tau_{\text{er}}$). Strong adaptation of the ISI is most plausibly realized by a large net loss unless replenishment is assumed to be extremely slow. We observe that the SCC $\rho_{1}$ is maximized when ΔT is long except for very short transients. The SCC depends nonmonotonically on $n_{\text{tr}}$. This has already been argued above but is shown in Fig. 10, *A*_3_ and *B*_3_ to be true over a wide range of transient statistics and in both firing regimes.

### Interspike interval statistics of stimulated HEK cells

In this section, we study to which extent our model is able to reproduce experimental sequences of ISIs observed in HEK cells subject to the onset of a constant stimulation as described in (4,12). As in the first part of this paper we select from the available data only the ISI sequences that become stationary according to visual inspection (29/36 sequences). In addition, we require that spikes can be well distinguished also during the transient (24/29 sequences). For the remaining 24 sequences, in a first step, four experimental output statistics are determined: the first interval $T_{0}$, the number of transient intervals $n_{\text{tr}}$, the stationary interval $T_{\infty }$, and the stationary coefficient of variation $C_{V}$. To determine the output statistics we fit the experimental ISI sequence $\left\{T_{i}\right\}$ by the exponential function Eq. 7 and obtain $T_{0}$, $n_{\text{tr}}$, and $T_{\infty }$ as fit parameters. We use the curve_fit function of the SciPy module (71) with the additional condition that all fitting parameters are positive. It is important to note that we previously used Eq. 7 to fit sequences of *mean* intervals $\left\{\left\langle T_{i}\right\rangle \right\}$, whereas now we fit single realizations of experimental sequences of intervals $\left\{T_{i}\right\}$. Next, the first $2n_{\text{tr}}$ (rounded up) intervals are truncated from the sequence of ISIs, and the remaining intervals are used to calculate the fourth parameter, the coefficient of variation of the stationary ISI, $C_{V}$. In a second step, we use a minimization algorithm to determine the four model parameters *τ*, *p*, $\tau_{\text{er}}$, and *ε* such that the model reproduces the four output statistics within a certain tolerance (see Appendix fit procedure for stimulated HEK cells).

**Figure 11:**
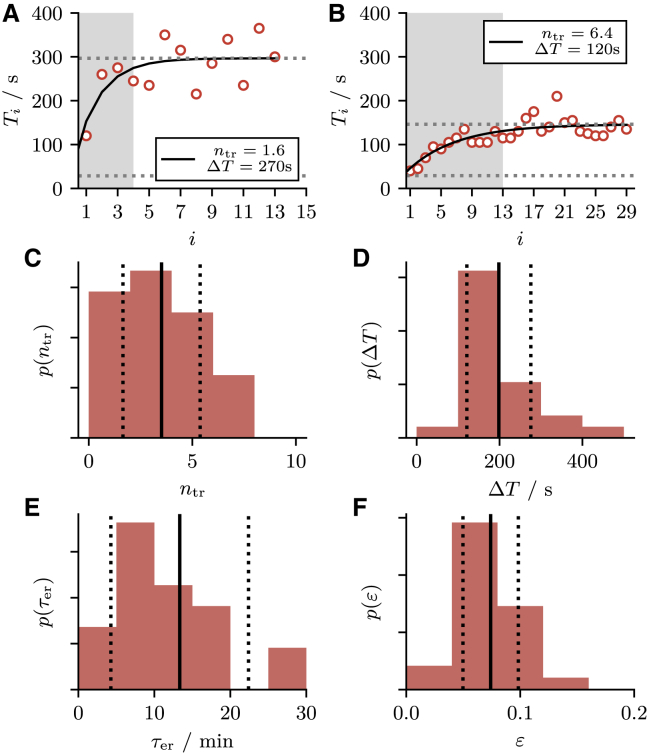
Transient ISIs of stimulated HEK cells. (*A* and *B*) An exemplary sequence of ISIs $\left\{T_{i}\right\}$ (*red circles*) with small (large) number of transient intervals $n_{\text{tr}}$ and large (small) cumulative refractory time ΔT. The sequence is fit by $T_{\infty }-\left(T_{\infty }-T_{0}\right)\exp\left(-i/n_{\text{tr}}\right)$ (*black line*) from which $n_{\text{tr}}$ and ΔT are determined as fit parameters. (*C* and *D*) The histograms of $n_{\text{tr}}$ (with $\mu \left(n_{\text{tr}}\right)=3.5$ and $\sigma \left(n_{\text{tr}}\right)=1.9$) and ΔT (with μ(ΔT)=200s and σ(ΔT)=80s) over all cells that have been analyzed. Vertical solid black lines indicate the mean, dotted lines indicate the standard deviation. For each cell, we use a two-step fit procedure as described in the Appendix to find the model parameters that reproduce the experimental statistics. (*E* and *F*) The histograms of $\tau_{\text{er}}$ (with $\mu \left(\tau_{\text{er}}\right)=800\;s$ and $\sigma \left(\tau_{\text{er}}\right)=500\;s$) and *ε* (with μ(ε)=0.07 and σ(ε)=0.02). To see this figure in color, go online.

Two sequences of ISIs (*red circles*) and the corresponding fits (*black line*) are shown in Fig. 11, *A* and *B*. The gray area indicates the intervals that are associated with the transient and that we truncate to calculate the CV. It should be noted that the sequences are subject to considerable cell-to-cell variability. For example, the sequence in Fig. 11 *A* has a small number of transient intervals $n_{\text{tr}}$ but a large cumulative refractory period ΔT, whereas the opposite is true for the sequence in Fig. 11 *B*. The full variability of the fit parameters becomes apparent in the histograms of $n_{\text{tr}}$ and ΔT shown in Fig. 11, *C* and *D*. Here, the solid and dotted black lines indicate mean $\mu \left(y\right)=\sum_{i=1}^{n}y_{i}/n$ and standard deviation $\sigma \left(y\right)={\left[\sum_{i=1}^{n}{\left(y_{i}-\mu \left(y\right)\right)}^{2}/\left(n-1\right)\right]}^{1/2}$, respectively (*y* is a dummy variable). We find that both the number of transient intervals $n_{\text{tr}}$ ($\mu \left(n_{\text{tr}}\right)=3.5$, $\sigma \left(n_{\text{tr}}\right)=1.9$) and the cumulative refractory period ΔT (μ(ΔT)=200s, σ(ΔT)=80s) vary significantly across different HEK cells.

Our fit procedure yields for every combination of output statistics a set of model parameters *τ*, *p*, $\tau_{\text{er}}$, and *ε*. The parameters *τ* and *p* determine whether the model operates in the mean-driven or excitable regime. It turns out that to reproduce the output statistics of stimulated HEK cells, our model is, for all data sets, poised in the mean-driven regime. Furthermore, we show in more detail the statistics of the extracted model parameters $\tau_{\text{er}}$ and *ε* in Fig. 11, *E* and *F* that also exhibit a strong variability. Regarding the average values we note that the mean timescale obtained from the fit procedure $\mu \left(\tau_{\text{er}}\right)=800$ s is rather long while the mean value of the depletion amplitude μ(ε)=0.07 is small.

**Figure 12:**
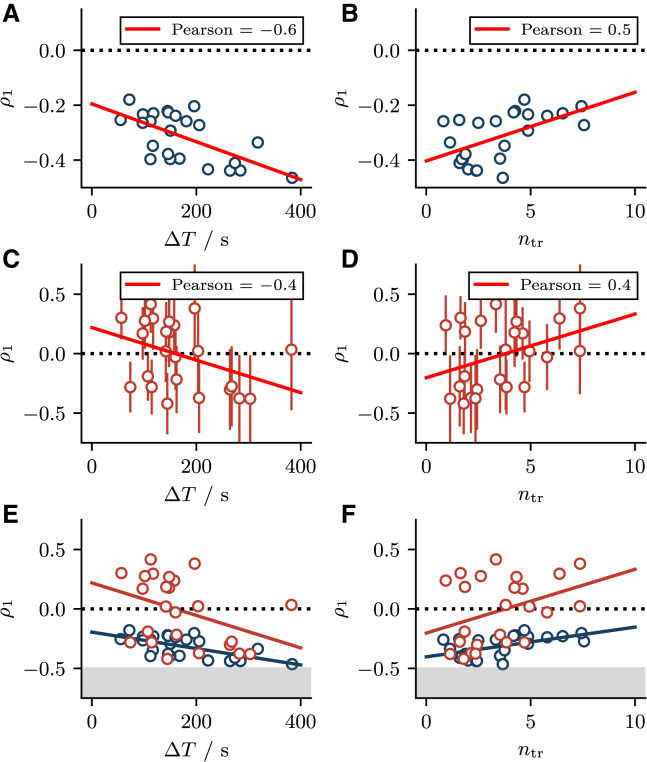
Stationary over transient statistics for HEK cells and fitted model. (*A* and *B*) The SCC $\rho_{1}$ as a function of the cumulative refractory period ΔT and number of transient intervals $n_{\text{tr}}$ obtained from simulations with model parameters as obtained from the fit procedure. Red lines indicate the linear regression lines with the Pearson correlation coefficient given in the legend. (*C* and *D*) The same relations and regression lines, with $\rho_{1}$ calculated from the stationary part of the experimental ISI sequence and with error bars. (*E* and *F*) Direct comparison of SCC calculated from simulated and experimental sequences. Gray areas highlight the region $\rho_{1}<-1/2$ that is inaccessible if $\rho_{k}$ decays monotonically. To see this figure in color, go online.

For each set of model parameters, we carry out long simulations and calculate the SCC $\rho_{1}$ as a function of (the likewise measured) ΔT (Fig. 12 *A*) and $n_{\text{tr}}$ (Fig. 12 *B*); we recall that the correlation coefficient of the experimental data was not part of our fitting routine. Furthermore, we note that the variability of $\rho_{1}$ does not stem from a simulation error but reflects the variability of the model parameters. According to our discussion in the previous section, for small values of *ε* (as determined in our fit) we expect to find a stronger interval correlation $\rho_{1}$ for a larger cumulative refractory period ΔT—a trend confirmed by the (*red*) regression line. The observed dependence of $\rho_{1}$ on $n_{\text{tr}}$ indicates that the latter mainly varies due to changes in the depletion amplitude *ε* but not so much due to variations in $\tau_{\text{er}}$. This is consistent with our observation in Fig. 9, *A*_2_ and *B*_2_, i.e., that, in the relevant parameter regime, longer transients typically concur with weaker interval correlations in the stationary state.

How do the correlation coefficients simulated in our model at the parameter sets relate to $\rho_{1}$ obtained from the experimental data directly? First of all, the experimental data are much more limited in extent (17 intervals on average) than our simulation data (at least 10,000 intervals at each parameter set) and therefore the experimental values of $\rho_{1}$ have a large numerical error (see large error bars in Fig. 12, *C* and *D* and (56) for an extensive discussion on sampling errors). Secondly, $\rho_{1}$ from the data is in a sizable fraction of cases positive, a feature that cannot be explained in the framework of our model. Similar between model and experimental data are the general trends of the relations between the SCC $\rho_{1}$ and ΔT (negative correlation) and between the SCC $\rho_{1}$ and $n_{\text{tr}}$ (positive correlation), i.e., we have similar regression lines in Fig. 12, *A* and *C* and in Fig. 12, *B* and *D*. Note that interval correlations have been shown to vanish when averaged over multiple cells of the same type (12,38). This result is consistent with the correlation coefficients from experiments presented here, which also vanish when averaged over all cells $\left\langle \rho_{1}\right\rangle =-0.01\pm 0.06$. Only when the SCCs are plotted against the correct parameters does a trend emerge.

To explain the observed positive ISI correlations, we could think of a weak nonstationarity that can cause subsequences of intervals to deviate similarly from the mean interval. Indeed, for our data we calculated the SCC over the last $n-2n_{\text{tr}}$ intervals, which were assumed to be stationary. However, as shown in Fig. 11, *A* and *B*, there are still systematic deviations from the stationary mean value after the first $2n_{\text{tr}}$ intervals (difference between the *black line* and the *upper dotted line*). This trend is weak but may play a role if the variance of the intervals is small. From a biophysical point of view, it could also be that there are other slow feedback processes in the generation of Ca^2+^ spikes that we have not included in our model (Ca^2+^ currents between cytosol and mitochondria might be a candidate for such a process). When we compare the model and simulation data in a single plot (Fig. 12, *E* and *F*) we observe a striking bimodality of the experimental SCC: the SCCs fall into two categories, one for which the SCC is negative and our model reproduces it well, and one for which the SCC is positive, which cannot be explained by our model. This could indicate that, for some cells, the depletion of the ER is indeed the dominating process that determines the interval correlations, while for other cells other processes that we have not accounted for are more relevant.

## Summary and discussion

We added a slow variable, i.e., the lumenal Ca^2+^ concentration, to our description of Ca^2+^ spike generation by integrate-and-fire type models in this study. Initially, at the onset of spiking, the amount of Ca^2+^ released during a spike is not completely replenished in between spikes. The ER is depleted partially over multiple spikes and the ISI is lengthened until the amount of Ca^2+^ lost during spiking and the replenishment between spikes balance each other. This phenomenon is known as spike-frequency adaptation in the theory of neural spike generation. While the importance of ER or total cell Ca^2+^ has been extensively studied both experimentally (29,30,31,32) and theoretically (33,34,35), to the best of our knowledge no stochastic model has addressed the implications of the slow decline of ER Ca^2+^ concentration in terms of transient and stationary spiking statistics. We have filled this gap.

The duration of the transient toward the stationary state can be expressed either by the effective time $\tau_{\text{eff}}$ (defined by Eq. 28) or by the number of transient ISIs $n_{\text{tr}}$ (defined by Eq. 7). The longer it takes to replenish the ER, the longer is the transient (Figs. 7 and 8). Remarkably the duration of the initial transient is always shorter than the timescale at which the ER is replenished by SOCE $\tau_{\text{eff}}$¡ $\tau_{\text{er}}$. The larger the fraction of ER content lost during a spike *ε*, the larger is the adaptation of the ISI ΔT, and the shorter is the transient (Figs. 7 and 8). If replenishment is slow, long transients with little adaptation arise from small fractions of ER content lost during a spike.

The partial depletion of the ER entails negative correlation between subsequent ISIs. If an ISI is very long, SOCE refills the ER well. The following spike will release the fraction *ε* of ER content, but since the ER is well filled before the spike, the remaining content after the spike is still high and the spike generation probability is high. Thus, this ISI is likely to be shorter than the previous one. The effect of an ISI on its successor is the strongest if the adaptation ΔT is large and the number of transient ISIs $n_{\text{tr}}$ is small (Fig. 12). This is the case if *ε* > 0.1 holds, i.e., if the ER is substantially depleted during a single spike.

Several studies suggested that the stationary coefficient of variation CV of the ISI sequence is set by the timescale of recovery from the negative feedback terminating spikes, but is robust against changes in other cellular parameters. Since the type of negative feedback is cell-type and agonist specific, so is the CV (4,37,39,40,72,73). Here, this timescale is $\tau_{\text{er}}$. CV values observed in different cell types range from 0.17 for hepatocytes stimulated with vasopressin to 0.94 for astrocytes (4,37,39,40,72,73). These CVs are compatible with our model when operating in the excitable regime. Our study adds a novel aspect: the relative amplitude of the negative feedback during a single spike, determined by *ε*, also affects the CV. This finding is in line with the above statements, since it is also a property of the type of feedback and thus cell-type specific.

Finally, we have fit our model to experimentally observed ISI sequences and used the obtained parameter sets to test whether the observed interval correlations and the observed relations between transient and stationary statistics are reproduced by our model. We note that, to reproduce the rather regular spike trains generated by stimulated HEK cells, our model is always in the mean-driven regime. This is due to the fact that the fluctuations of the cytosolic Ca^2+^ concentration, caused by the random release of Ca^2+^ by the IP_3_R clusters, are often rather weak. This is a property that our model shares with other spatial averaging approaches (74). As a consequence, fluctuation-driven spiking is observed only in the vicinity of the bifurcation.

Fig. 10 illustrates the range of ΔT and $n_{\text{tr}}$ values compatible with our theory. Almost all experimental values are within that range. Moreover, we find that the general trends between the correlation coefficient on the one hand, and the cumulative refractory period ΔT and the number of transient intervals $n_{\text{tr}}$, on the other hand, are well reproduced by the model. At the same time, however, we observe a number of positive interval correlations in the experimental data that cannot be explained by our model. Fig. 12 seems to indicate, that the observed correlation coefficients that are negative are well reproduced, while the coefficients that are positive are not. It is difficult to conclude whether this is due to poor statistics computed from short ISI sequences or to other slow feedback mechanisms in HEK cells that we have not accounted for in the model.

### Limitations of the model

In our simulation we used a reset value for $c_{i}$, which is determined by $c_{\text{er}}$ and the parameter $c_{i}^{0}$. This corresponds in the experiment to the concentration attained at the end of the refractory period after the spike. That entails a minimal $c_{i}$ in between spikes decreasing on average as the cell adapts to the spiking state. This is a valid description of the subthreshold behavior of some cell types (4,22,36,75), but other cell types show more variability. The reset value affects the ISI statistics and thus is worth to be investigated in more detail in future studies.

Several observations led to the conclusion that spiking cells operate rather in the excitable regime than in a mean-driven regime. Theoretical studies showed the lack of local oscillatory dynamics providing global oscillations (76) since local concentrations are outside the dynamic range of the Ca^2+^ feedback to the channel (77,78,79). Experiments confirmed the lack of local oscillatory dynamics (37). The strong sensitivity of the average ISI to the strength of spatial coupling very much supports the idea of spike generation by Ca^2+^ wave nucleation in an excitable regime rather than a cellular limit cycle oscillation (12). The steep spatial $c_{i}$-gradients around releasing channels and clusters (77,78,79) preclude local oscillatory dynamics and cause the sensitivity to spatial coupling. Here, we neglect these gradients. Instead, we have assumed that Ca^2+^ is homogeneously distributed in both the cytosol and the ER. Spatially averaged cytosolic Ca^2+^ concentrations are in the dynamic range of the Ca^2+^ feedback to the channel, but are not the Ca^2+^ concentrations experienced by the regulatory binding sites on the channel molecule (77,78,79). Voorsluijs et al. simulate spiking as limit cycle oscillations with spatially averaged Ca^2+^ concentrations (80). The spatial average excludes the wave nucleation mechanism but spikes may arise from noise in an excitable regime (80,81).

**Figure 13:**
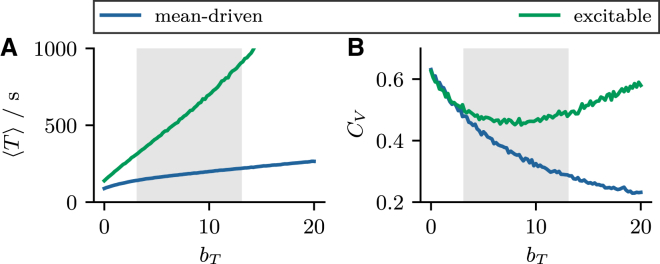
A CV-based test of the dynamic regime. (*A* and *B*) The mean ⟨T⟩ and CV $C_{V}$ of the ISI over the total buffer concentration $b_{T}$. In the mean-driven regime (*blue lines*), $C_{V}$ decreases monotonically with $b_{T}$ and saturates at 0. In the excitable regime (*green line*), $C_{V}$ exhibits a minimum before saturating at 1. The asymptotic ($b_{T}\to \infty$) behavior of the CV allows to distinguish the firing regimes. Parameters: K=5, τ=1s, ε=0.03, $\tau_{\text{er}}=300\;s$, $k^{-}=1s^{-1}$, $k^{+}=1s^{-1}$ and (mean driven) $p=1.05p_{\text{bif}}\approx 0.136$, (excitable) $p=0.95p_{\text{bif}}\approx 0.123$. To see this figure in color, go online.

Our model cannot describe Ca^2+^ concentration gradients because it is not spatially extended. As a consequence, it is difficult to decide whether stimulated HEK cells truly operate in the mean-driven regime, as suggested by our model, because the parameters that determine the firing regime were found to be very close to the bifurcation line and crucially dependent on the details of the model. Therefore, we propose a simple experimental test using a fast Ca^2+^ buffer to determine whether a cell is mean driven or fluctuation driven. In Fig. 13, we show the mean and CV as a function of the total concentration of a fast Ca^2+^ buffer, which can be easily controlled in the experiment. As we have shown in the Appendix variation of noise intensity by a fast Ca2+ buffer, adding a fast buffer effectively reduces the noise intensity according to ${\hat{D}}_{x}\left(c_{i}\right)=\beta D_{x}\left(c_{i}\right)$ with $\beta \approx 1/\left(1+b_{T}/K^{*}\right)<1$. In the mean-driven regime, the ISI variability decreases monotonically and spike formation becomes a deterministic process for large buffer concentrations, $\lim_{b_{T}\to \infty }C_{V}=0$. In the fluctuation-driven regime, the coefficient of variation exhibits a coherence resonance minimum (81,82) as a function of the buffer concentration and becomes a Poisson process in the limit $\lim_{b_{T}\to \infty }C_{V}=1$. Moreover, the coherence resonance minimum is rather flat and the CV does not change much over a wide range of buffer concentrations (*green line* in *gray area* in Fig. 13 *B*); in the same range, the mean interval changes drastically with $b_{T}$ (*green line* in *gray area* in Fig. 13 *A*). Taken together, this is in line with the experimental observation of a constant CV over a wide range of mean intervals in stimulated HEK cells loaded with different levels of BAPTA (12). This suggests that stimulated HEK cells operate in the fluctuation-driven regime. A more definite test would explore the spike statistics in the limit of even larger buffer concentrations.

## Author contributions

Conceptualization, B.L., M.F., and M.F.; methodology, L.R. and B.L.; software, L.R.; investigation, L.R. and B.L.; data curation, L.R.; writing – original draft, L.R. and B.L.; writing – review & editing, M.F.; visualization, L.R.; validation, B.L.; supervision, B.L. and M.F.; project administration, B.L. and M.F. All authors reviewed the results and approved the final version of the manuscript.

## Figures and Tables

**Figure 14:**
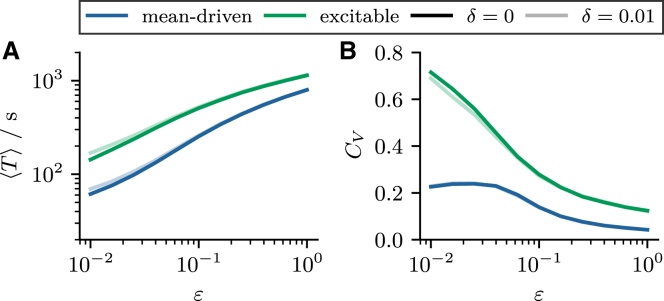
Effect of subthreshold ER depletion on spiking statistics. (*A*) Comparison of the mean ISI of the model used in the main part (Eq. 1) with the mean of a model variant in which the subthreshold Ca^2+^ released from the ER into the cytosol is included in the dynamics not only of $c_{i}$ but also of $c_{\text{er}}$. This amounts to extending the equation governing the dynamics of $c_{\text{er}}$ by Eq. 54. Similarly, (*B*) compares the CVs. Parameters: (mean driven) τ=5s, p=0.015, $\tau_{\text{er}}=300\;s$; (excitable) τ=1s, p=0.06, $\tau_{\text{er}}=300\;s$. To see this figure in color, go online.

**Figure 15:**
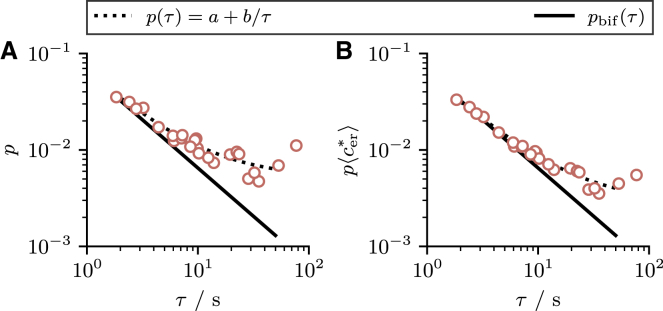
Renewal model system parameters. (*A*) The two system parameters *τ* and *p* obtained from the fit procedure are highly correlated and follow the relationship p=a+b/τ (*dotted line*). This is because the model operates in the vicinity of the bifurcation (*black line*). (*B*) The model still operates in the mean-driven regime when stationary mean value $\left\langle c_{\text{er}}^{*}\right\rangle$ is taken into account. To see this figure in color, go online.

## Appendix

### Subthreshold Ca^2+^ dynamics

To incorporate the ER Ca^2+^ concentration into our framework, we need to derive dynamical equations governing the cytosolic Ca^2+^ concentration ${\left[{\text{Ca}}^{2+}\right]}_{i}$ and ER Ca^2+^ concentration ${\left[{\text{Ca}}^{2+}\right]}_{\text{er}}$. As in the first part of this paper, the subthreshold dynamics of ${\left[{\text{Ca}}^{2+}\right]}_{i}$ are determined by two Ca^2+^ currents:

(37) $$\frac{d}{dt}{\left[{\text{Ca}}^{2+}\right]}_{i}=J_{\text{lin}}+J_{\text{puff}}\text{,}$$

where $J_{\text{lin}}$ is an IP_3_-independent linearized current, which subsumes all active and passive currents into the cytosol, and $J_{\text{puff}}$ is the IP_3_-dependent nonlinear puff current, which describes the release of Ca^2+^ from the ER into the cytosol through IP_3_ receptor channels. We regard $J_{\text{lin}}$ as a sum of currents across the ER membrane and the cell membrane

(38) $$J_{\text{lin}}=J_{\text{er},i}+J_{\text{ext},i}$$

(39) $$J_{\text{er},i}=-\frac{{\left[{\text{Ca}}^{2+}\right]}_{i}}{\tau_{s}}+\frac{V_{\text{er}}}{V_{i}}\frac{{\left[{\text{Ca}}^{2+}\right]}_{\text{er}}}{\tau_{l}}$$

(40) $$J_{\text{ext},i}=-\frac{{\left[{\text{Ca}}^{2+}\right]}_{i}-{\left[{\text{Ca}}^{2+}\right]}_{i}^{0}}{\tau_{p_{m}}}\text{.}$$

The first term in Eq. 39 describes the active reuptake of Ca^2+^ from the cytosol into the ER by SERCA pumps with timescale $\tau_{s}$ (here appearing as a loss term). The second term in Eq. 39 captures the passive leakage from the ER into the cytosol with timescale $\tau_{l}$ and volume ratio between ER and cytosol $V_{\text{er}}/V_{i}$. Note that, strictly speaking, this term should depend on the difference ${\left[{\text{Ca}}^{2+}\right]}_{\text{er}}-{\left[{\text{Ca}}^{2+}\right]}_{i}$, which we approximate by ${\left[{\text{Ca}}^{2+}\right]}_{\text{er}}$ because ${\left[{\text{Ca}}^{2+}\right]}_{\text{er}}\gg {\left[{\text{Ca}}^{2+}\right]}_{i}$ (44,45,46). The term in Eq. 40 includes a number of active and passive Ca^2+^ currents across the plasma membrane and is characterized by an effective timescale $\tau_{p_{m}}$. For more detailed physiological models, see (33,74,83,84).

In the steady state, both net currents in Eq. 38 have to vanish, i.e., $J_{\text{ext},i}=0$ and $J_{\text{er},i}=0$. The former condition determines the steady-state concentration in the cytosol, ${\left[{\text{Ca}}^{2+}\right]}_{i}={\left[{\text{Ca}}^{2+}\right]}_{i}^{0}$; the latter yields the steady-state concentration in the ER ${\left[{\text{Ca}}^{2+}\right]}_{\text{er}}={\left[{\text{Ca}}^{2+}\right]}_{\text{er}}^{0}$, which is related to ${\left[{\text{Ca}}^{2+}\right]}_{i}^{0}$ by

(41) $${\left[{\text{Ca}}^{2+}\right]}_{\text{er}}^{0}={\left[{\text{Ca}}^{2+}\right]}_{i}^{0}\frac{\tau_{l}}{\tau_{s}}\frac{V_{i}}{V_{\text{er}}}\text{.}$$

As in the first part of this paper, the currents in Eq. 38 can be combined into a single term

(42) $$J_{\text{lin}}=-\frac{{\left[{\text{Ca}}^{2+}\right]}_{i}-{\left[{\text{Ca}}^{2+}\right]}_{i}^{*}}{\tau }$$

with the timescale

(43) $$\tau =\frac{\tau_{s}\tau_{p_{m}}}{\tau_{p_{m}}+\tau_{s}}$$

and the ${\left[{\text{Ca}}^{2+}\right]}_{\text{er}}$-dependent concentration

(44) $${\left[{\text{Ca}}^{2+}\right]}_{i}^{*}=\left(\frac{{\left[{\text{Ca}}^{2+}\right]}_{i}^{0}}{\tau_{p_{m}}}+\frac{V_{i}}{V_{\text{er}}}\frac{{\left[{\text{Ca}}^{2+}\right]}_{\text{er}}}{\tau_{l}}\right)\tau \text{.}$$

Equation 44 can be recast using Eqs. 41 and 43:

(45) $${\left[{\text{Ca}}^{2+}\right]}_{i}^{*}={\left[{\text{Ca}}^{2+}\right]}_{i}^{0}\left[\frac{\tau_{s}}{\tau_{p_{m}}+\tau_{s}}+\frac{\tau_{p_{m}}}{\tau_{p_{m}}+\tau_{s}}\frac{{\left[{\text{Ca}}^{2+}\right]}_{\text{er}}}{{\left[{\text{Ca}}^{2+}\right]}_{\text{er}}^{0}}\right]\text{.}$$

In agreement with other theoretical studies (74,84,85), we can further simplify this result. We assume that the leakage across the plasma membrane is much slower than the reuptake by the SERCA pumps, i.e., $\tau_{p_{m}}\gg \tau_{s}$, implying that the Ca^2+^ current across the plasma membrane is neglected. This assumption leads to a linear relation between ${\left[{\text{Ca}}^{2+}\right]}_{i}^{*}$ and ${\left[{\text{Ca}}^{2+}\right]}_{\text{er}}$:

(46) $${\left[{\text{Ca}}^{2+}\right]}_{i}^{*}={\left[{\text{Ca}}^{2+}\right]}_{i}^{0}\frac{{\left[{\text{Ca}}^{2+}\right]}_{\text{er}}}{{\left[{\text{Ca}}^{2+}\right]}_{\text{er}}^{0}}\text{.}$$

Finally, we insert Eq. 46 into 42 to obtain:

(47) $$J_{\text{lin}}=-\frac{\left({\left[{\text{Ca}}^{2+}\right]}_{i}-{\left[{\text{Ca}}^{2+}\right]}_{i}^{0}{\left[{\text{Ca}}^{2+}\right]}_{\text{er}}/{\left[{\text{Ca}}^{2+}\right]}_{\text{er}}^{0}\right)}{\tau }\text{.}$$

Turning to the puff current we note that, as a diffusive current, $J_{\text{puff}}$ is determined by the concentration gradient between cytosol and ER, ${\left[{\text{Ca}}^{2+}\right]}_{\text{er}}-{\left[{\text{Ca}}^{2+}\right]}_{i}\approx {\left[{\text{Ca}}^{2+}\right]}_{\text{er}}$, by the total number of open channels, $\sum_{k=1}^{K}x_{k}\left(t\right)$, and by a permeability-like parameter $\hat{p}$:

(48) $$J_{\text{puff}}=\hat{p}{\left[{\text{Ca}}^{2+}\right]}_{\text{er}}\sum_{k=1}^{K}x_{k}\left(t\right)\text{.}$$

Using the two currents and introducing nondimensional variables, $c_{i}={\left[{\text{Ca}}^{2+}\right]}_{i}/K_{\text{act}}$ and $c_{\text{er}}={\left[{\text{Ca}}^{2+}\right]}_{\text{er}}/{\left[{\text{Ca}}^{2+}\right]}_{\text{er}}^{0}$, yields the first line of Eq. 1:

(49) $${\dot{c}}_{i}=-\left(c_{i}-c_{i}^{0}c_{\text{er}}\right)/\tau +pc_{\text{er}}\sum_{k=1}^{K}x_{k}\left(t\right)$$

with $p=\hat{p}{\left[{\text{Ca}}^{2+}\right]}_{\text{er}}^{0}/K_{\text{act}}$.

For the dynamics of the ER Ca^2+^ concentration, we distinguish a replenishment term and a depletion term:

(50) $$\frac{d}{dt}{\left[{\text{Ca}}^{2+}\right]}_{\text{er}}=J_{\text{rep}}+J_{\text{dep}}\text{.}$$

First, in the absence of a spike $t\neq t_{i}$, we assume that the ER Ca^2+^ concentration is replenished exponentially by SOCE (86). SOCE is a process in which the depletion of the ER causes the formation of ER-PM junctions (87,88,89) and a slow Ca^2+^ current across the plasma membrane through Orai1 Ca^2+^ channels. As an approximation, we assume that these junctions allow SERCA pumps to relay the slow local Ca^2+^ influx from the extracellular medium directly into the ER (28). We note that computational studies have suggested that the cytosolic Ca^2+^ concentration is not entirely unaffected by SOCE due to the rapid diffusion of Ca^2+^ (90), an effect we neglect here. In our model, SOCE directly affects ER Ca^2+^ concentration:

(51) $$J_{\text{rep}}=-\left({\left[{\text{Ca}}^{2+}\right]}_{\text{er}}-{\left[{\text{Ca}}^{2+}\right]}_{\text{er}}^{0}\right)/\tau_{\text{er}}\text{.}$$

Second, when a spike is fired at $t=t_{i}$, the concentration is depleted immediately according to

(52) $$J_{\text{dep}}=-\varepsilon {\left[{\text{Ca}}^{2+}\right]}_{\text{er}}\delta \left(t-t_{i}\right)\text{.}$$

This term describes the difference between the ER Ca^2+^ concentration before and after the spike and, thus, the net loss of Ca^2+^ from the ER during the spike. The delta function enters because, in the IF framework, the spike is a point in time, so that both the release and the reuptake of Ca^2+^ during the rising and falling phases of the spike are described as instantaneous. Using Eq. 51 and Eq. 52 in Eq. 50 and dividing both the left- and right-hand side by ${\left[{\text{Ca}}^{2+}\right]}_{\text{er}}^{0}$ yields the second line of Eq. 1, i.e., the dynamics of the above-introduced nondimensional variable $c_{\text{er}}$:

(53) $${\dot{c}}_{\text{er}}=\frac{\left(c_{\text{er}}-1\right)}{\tau_{\text{er}}}-\varepsilon c_{\text{er}}\sum_{i}\delta \left(t-t_{i}\right)\text{.}$$

We note that the Ca^2+^ currents across the ER membrane that contribute to the subthreshold dynamics of the cytosolic Ca^2+^ concentration should in principle also contribute to the dynamics of the ER Ca^2+^ concentration with a reversed sign and a prefactor that accounts for the volume ratio. Carrying out all the steps as before, but taking into account the exact balance of currents across the ER membrane, will lead to an additional term on the r.h.s. of Eq. 53:

(54) $$-\delta \left(-\left(c_{i}-c_{i}^{0}c_{\text{er}}\right)/\tau +pc_{\text{er}}\sum_{k=1}^{K}x_{k}\left(t\right)\right)\text{.}$$

The prefactor $\delta =\left(V_{i}/V_{\text{er}}\right)\left(K_{\text{act}}/{\left[{\text{Ca}}^{2+}\right]}_{\text{er}}^{0}\right)$ is of the order of 0.001−0.01 (using $V_{i}/V_{\text{er}}\approx 10$ (91), $K_{\text{act}}\approx$ 100 *μ*m (92), and ${\left[{\text{Ca}}^{2+}\right]}_{\text{er}}^{0}\approx$ 100–1000 *μ*m (44,93)). The term in the parentheses is equal to the r.h.s. of the $c_{i}$ dynamics and its mean value can therefore be estimated to be $\left(c_{T}-c_{R}\right)/\left\langle T\right\rangle$, which is of the same order of magnitude as the replenishment term in Eq. 53, $\left(c_{\text{er}}-1\right)/\tau_{\text{er}}$. Multiplied by the small prefactor *δ*, the term can thus be neglected. To demonstrate that this is indeed the case, we augment Eq. 53 by the additional term Eq. 54. We compare the mean and CV of the stationary ISIs of the original model (δ=0) to the augmented version for a plausible value of δ=0.01. The spiking statistics hardly differ, irrespective of the value of *ε* and the firing regime (cf. Fig. 14).

### Average of the ER Ca^2+^ concentration

We have mentioned in the main part that averaging the governing differential equation for the ER Ca^2+^ concentration $c_{\text{er}}\left(t\right)$ gives rise to an unconditional and a conditional mean. Here, we relate these two mean values to one another.

To this end, we first recall the differential equation that governs the dynamics of $c_{\text{er}}\left(t\right)$:

(55) $${\dot{c}}_{\text{er}}=-\left(c_{\text{er}}-1\right)/\tau_{\text{er}}-\varepsilon c_{\text{er}}\sum_{i}\delta \left(t-t_{i}\right)$$

and take the stationary ensemble average to obtain:

(56) $$0=-\left(\left\langle c_{\text{er}}^{*}\right\rangle -1\right)/\tau_{\text{er}}-\varepsilon \left\langle c_{\text{er}}^{*}|t=t_{i}^{-}\right\rangle r_{0}\text{.}$$

This equation contains the conditional mean $\left\langle c_{\text{er}}^{*}|t=t_{i}^{-}\right\rangle$, which results from taking the average of $c_{\text{er}}\left(t\right)$ together with the spike train $\sum_{i}\delta \left(t-t_{i}\right)$. Here, the minus indicates the time just before the spike, i.e., $c_{\text{er}}\left(t_{i}^{-}\right)$ refers to the value before the instantaneous depletion.

To relate $\left\langle c_{\text{er}}^{*}\right\rangle$ and $\left\langle c_{\text{er}}^{*}|t=t_{i}^{-}\right\rangle$ we consider the replenishment and depletion of $c_{\text{er}}\left(t\right)$ separately. Between two spike times $t_{i}\leq t<t_{i+1}$ the governing differential equation for the ER Ca^2+^ concentration (Eq. 55) reduces to:

(57) $${\dot{c}}_{\text{er}}=-\left(c_{\text{er}}-1\right)/\tau_{\text{er}}\text{,}$$

and is solved by:

(58) $$c_{\text{er}}\left(t\right)=1-\left[1-c_{\text{er}}\left(t_{i}^{+}\right)\right]e^{-\left(t-t_{i}\right)/\tau_{\text{er}}}\text{,}$$

where $c_{\text{er}}\left(t_{i}^{+}\right)$ is the initial condition over the interval $T_{i}$. Note that when $t=t_{i+1}^{-}$ the variable $c_{\text{er}}\left(t\right)$ attains the value $c_{\text{er}}\left(t_{i+1}^{-}\right)$. This somewhat trivial observation allows to find a first relation between the two stationary conditional means

(59) $$\left\langle c_{\text{er}}^{*}|t=t_{i}^{-}\right\rangle =1-\left\langle \left[1-c_{\text{er}}\left(t_{i}^{+}\right)\right]e^{-\frac{T_{i}}{\tau_{\text{er}}}}\right\rangle \text{.}$$

At a spike time $t=t_{i}$ the ER is instantaneously depleted and a second relation between the stationary conditional mean values is readily obtained:

(60) $$\left\langle c_{\text{er}}^{*}|t=t_{i}^{+}\right\rangle =\left\langle c_{\text{er}}^{*}|t=t_{i}^{-}\right\rangle \left(1-\varepsilon \right)\text{.}$$

Finally, to make a relation to the unconditional mean we consider the temporal average:

(61) $$\left\langle c_{\text{er}}^{*}\right\rangle =\lim_{T\to \infty }\int_{0}^{T}dt\;c_{\text{er}}\left(t\right)$$

and split the integral at the spike times to involve the conditional mean values:

(62) $$\begin{array}{l} \left\langle c_{\text{er}}^{*}\right\rangle =\lim_{T\to \infty }\frac{1}{T}\sum_{T_{i}\in \left[0,T\right]}\int_{0}^{T_{i}}dt1-\left[1-c_{\text{er}}\left(t_{i}^{+}\right)\right]e^{-\left(t-t_{i}\right)/\tau_{\text{er}}}=\lim_{T\to \infty }\frac{1}{T}\sum_{T_{i}\in \left[0,T\right]}T_{i}-\tau_{\text{er}}\left[1-c_{\text{er}}\left(t_{i}^{+}\right)\right]\left[1-e^{-T_{i}/\tau_{\text{er}}}\right]\\ =1-\frac{\tau_{\text{er}}}{\left\langle T\right\rangle }\left(\left[1-\left\langle c_{\text{er}}^{*}|t=t_{i}^{+}\right\rangle \right]-\left\langle \left[1-c_{\text{er}}\left(t_{i}^{+}\right)\right]e^{-T_{i}/\tau_{\text{er}}}\right\rangle \right)\\ =1-\frac{\tau_{\text{er}}}{\left\langle T\right\rangle }\left(\left\langle c_{\text{er}}^{*}|t=t_{i}^{-}\right\rangle -\left\langle c_{\text{er}}^{*}|t=t_{i}^{+}\right\rangle \right)\\ =1-\varepsilon \frac{\tau_{\text{er}}}{\left\langle T\right\rangle }\left\langle c_{\text{er}}^{*}|t=t_{i}^{-}\right\rangle \text{.} \end{array}$$

To obtain the first line we have inserted the solution of $c_{\text{er}}\left(t\right)$ between two spikes $t_{i}\leq t<t_{i+1}$ according to Eq. 58. To get from the third line to the fourth line and from the fourth line to the fifth line, we used equations Eqs. 59 and 60, respectively. This provides an exact relation between the conditional and unconditional mean values but comes with the disadvantage of containing the mean of the stationary interval. An approximate relation can derive assuming that $c_{\text{er}}\left(t\right)$ is replenished slowly and over an ISI evolves according to:

(63) $$c_{\text{er}}\left(t\right)\approx c_{\text{er}}\left(t_{i}^{+}\right)+\frac{c_{\text{er}}\left(t_{i+1}^{-}\right)-c_{\text{er}}\left(t_{i}^{+}\right)}{T_{i}}t\text{.}$$

This relation ensures that at the beginning and end of the ISI $T_{i}$ the values $c_{\text{er}}\left(t_{i}^{+}\right)$ and $c_{\text{er}}\left(t_{i+1}^{-}\right)$ are taken. In this case the unconditional mean and conditional mean are related by

(64) $$\left\langle c_{\text{er}}^{*}\right\rangle \approx \frac{\left\langle c_{\text{er}}|t=t_{i}^{-}\right\rangle +\left\langle c_{\text{er}}|t=t_{i}^{+}\right\rangle }{2}\text{.}$$

Using again Eq. 60 yields:

(65) $$\left\langle c_{\text{er}}^{*}\right\rangle \approx \left\langle c_{\text{er}}^{*}|t=t_{i}^{-}\right\rangle \left(1-\frac{\epsilon }{2}\right)$$

the expression that we have used in the main part.

### Variation of noise intensity by a fast Ca^2+^ buffer

It is often difficult to decide whether a cell that generates a stochastic sequence of spike times is operating in the mean-driven or excitable regime. An accurate answer would require considering the cell in the deterministic limit, e.g., by properly increasing the cell size so that the number of IP_3_R clusters *K* is increased while the effect of a single puff on the Ca^2+^ concentration, determined by the parameter *p*, is simultaneously decreased so that pK=const. Of course, this is not experimentally feasible. However, it is possible to mimic the effect of increasing the cell by adding a fast Ca^2+^ buffer, such as BAPTA.

To demonstrate this, we consider a fast Ca^2+^ buffer B according to the reaction scheme:

(66) $${\text{Ca}}^{2+}+B\overset{k^{+}}{\underset{k^{-}}{⇌}}\text{CaB,}$$

where $k^{+}$ and $k^{-}$ are rate constants. In this case, the governing differential equations of our model become:

(67) $$\begin{array}{l} {\dot{c}}_{i}=-\left(c_{i}-c_{i}^{0}c_{\text{er}}\right)/\tau +j_{\text{puff}}\left(c_{i},c_{\text{er}}\right)-k^{+}c_{i}\left(b_{T}-c_{b}\right)+k^{-}c_{b}\\ {\dot{c}}_{\text{er}}=-\left(c_{\text{er}}-1\right)/\tau_{\text{er}}-\varepsilon c_{\text{er}}\sum_{i}\delta \left(t-t_{i}\right)\text{,}\\ {\dot{c}}_{b}=k^{+}c_{i}\left(b_{T}-c_{b}\right)-k^{-}c_{b}\\ \text{if}c_{i}\left(t\right)=c_{T}\to t_{i}=t\;\text{and}\;c_{i}\left(t\right)=c_{R},c_{b}\left(t\right)=c_{b}^{0}\left(c_{R}\right)\text{,} \end{array}$$

where $b_{T}=b+c_{b}$ is the total buffer concentration given by the sum of the free and Ca^2+^-bound buffer concentration *b* and $c_{b}$, respectively. The total buffer concentration $b_{T}$ is assumed to be constant. In an IF framework, a fast Ca^2+^ buffer requires an additional reset rule to ensure that $c_{b}$ is still in equilibrium with $c_{i}$ after the instantaneous reset. Specifically, $c_{b}$ is reset to the equilibrium value, $c_{b}^{0}\left(c_{i}\right)=c_{i}b_{t}/\left(K^{*}+c_{i}\right)$ with $K^{*}=k^{-}/k^{+}$.

To show that a fast Ca^2+^ buffer effectively reduces the noise intensity, it is useful to utilize the Langevin approximation of our model and the fast buffer approximation (94):

(68) $$\begin{array}{l} {\dot{c}}_{i}=\beta \left[-\left(c_{i}-c_{i}^{0}c_{\text{er}}\right)/\tau +p\mu \left(c_{i},c_{\text{er}}\right)+p\sqrt{2D\left(c_{i},c_{\text{er}}\right)}\xi \left(t\right)\right]\\ {\dot{c}}_{\text{er}}=-\left(c_{\text{er}}-1\right)/\tau_{\text{er}}-\varepsilon c_{\text{er}}\sum_{i}\delta \left(t-t_{i}\right)\text{,}\\ \text{if}c_{i}\left(t\right)=c_{T}\to t_{i}=t\text{and}c_{i}\left(t\right)=c_{R}\text{,} \end{array}$$

with $\beta ={\left(1+K^{*}b_{T}/{\left(K^{*}+c_{i}\right)}^{2}\right)}^{-1}$. Assuming that $K^{*}\gg c_{i}$ allows to approximate $\beta \approx {\left(1+b_{T}/K^{*}\right)}^{-1}$ and to introduce a new time $t^{\prime }=\beta t$. Because the white noise in the $c_{i}$-dynamics and the delta function in the $c_{\text{er}}$-dynamics are time-dependent functions, they must also be scaled when the new time $t^{\prime }$ is introduced accordingly to $\xi \left(t^{\prime }/\beta \right)=\sqrt{\beta }\xi \left(t^{\prime }\right)$ and $\delta \left(t^{\prime }/\beta \right)=\beta \delta \left(t^{\prime }\right)$. This yields:

(69) $$\begin{array}{l} \frac{dc_{i}}{dt^{\prime }}=-\left(c_{i}-c_{i}^{0}c_{\text{er}}\right)/\tau +p\mu \left(c_{i},c_{\text{er}}\right)+p\sqrt{2\hat{D}\left(c_{i},c_{\text{er}}\right)}\xi \left(t^{\prime }\right)\\ \frac{dc_{\text{er}}}{dt^{\prime }}=-\left(c_{\text{er}}-1\right)/{\hat{\tau }}_{\text{er}}-\varepsilon c_{\text{er}}\sum_{i}\delta \left(t^{\prime }-t_{i}^{\prime }\right)\text{,}\\ \text{if}\;c_{i}\left(t^{\prime }\right)=c_{T}\to t_{i}^{\prime }=t^{\prime }\;\text{and}\;c_{i}\left(t^{\prime }\right)=c_{R}\text{,} \end{array}$$

with the effective noise intensity $\hat{D}\left(c_{i},c_{\text{er}}\right)=\beta D\left(c_{i},c_{\text{er}}\right)$ and the effective timescale ${\hat{\tau }}_{\text{er}}=\beta \tau_{\text{er}}$. This means that when the dynamics are observed with respect to the new time $t^{\prime }$, the noise intensity is effectively reduced by a factor β<1. Note that at the same time the timescale of the adjustment is reduced.

### Fit procedure for stimulated HEK cells

Here, we detail the minimization algorithm by which the model parameters are determined. We have already explained that, for each experimental ISI sequence, four output statistics are determined, namely the first interval $T_{0}$, the number of transient intervals $n_{\text{tr}}$, the stationary interval $T_{\infty }$, and the stationary coefficient of variation $C_{V}$. These statistics are used to estimate the four model parameters *τ*, *p*, $\tau_{\text{er}}$, and *ε*. We note that the full model comprises a larger number of parameters, but we keep those affecting the IP_3_R model fixed.

To determine the four parameters, we use the Nelder-Mead method (95) to minimize the loss function $f\left(y_{1},\ldots ,y_{4}\right)=\sum_{i}\left|y_{i}-{\hat{y}}_{i}\right|/y_{i}$, where $y_{i}\in \left[T_{0},n_{\text{tr}},T_{\infty },C_{V}\right]$ are the target statistics obtained from the fit procedure described in the main part and ${\hat{y}}_{i}\in \left[\left\langle T_{0}\right\rangle ,n_{\text{tr}},\left\langle T\right\rangle ,C_{V}\right]$ are the output statistics of the model. Because the model is stochastic, the output statistics (obtained from finite simulations) are also subject to a measurement error. To improve our estimate of the output statistics we use the numerically more efficient Langevin approximation Eq. 8.

Unfortunately, this is not sufficient to solve the minimization problem in a reasonable time. Therefore, we split the four-parameter minimization problem into two two-parameter minimization problems, which are solved iteratively until the interval statistics of the experiment and the model match. This essentially amounts to first determining the parameters of the nonadaptive model, *τ* and *p* (Part I, Eq. 2), and then the remaining parameters of the adaptive model, $\tau_{\text{er}}$ and *ε*. We recall that the first interval of the adaptive model is statistically equivalent to the ISI of the nonadaptive model because the ER Ca^2+^ concentration in the nonadaptive model is fixed to $c_{\text{er}}\left(t\right)=1$ for all *t*, which is also the case for the adaptive model before the first spike.

In a first step, we use the nonadaptive model to determine the parameters *τ* and *p* such that the mean and CV of the ISI of the nonadaptive model matches the fit parameter $T_{0}$ and $C_{V}$ from the experimental sequence. In a second step, we determine the remaining two model parameters $\tau_{\text{er}}$ and *ε* of the adaptive model. To this end, we require that the mean of the stationary sequence matches the output statistics $T_{\infty }$ and that the number of transient intervals, obtained from fitting Eq. 7 to the simulated sequence of mean intervals $\left\langle T_{i}\right\rangle$, matches the experimentally observed number of transient intervals $n_{\text{tr}}$.

The condition we used in the first step essentially required the CV of the first interval in the model $C_{V,0}$ to match the CV of the stationary interval in the experiment $C_{V,\infty }^{\text{HEK}}$. Since the CV in the model depends on the adaptation, this means that the stationary CV of the model $C_{V,\infty }$ does not agree with the stationary CV of the experiment. We therefore use an iterative procedure and change the target CV, denoted $C_{V,0}^{*}$ and used in the first step, based on the deviation between the stationary CVs of the model and the experiment according to $C_{V,0}^{*}\to C_{V,0}^{*}+\alpha \left(C_{V,\infty }^{\text{HEK}}-C_{V,\infty }\right)$. We find that for α=1 one to three iterations are sufficient to reproduce the experimental CV.

The resulting parameter pairs *τ* and *p* are shown in Fig. 15 *A* and closely follow the relation p=a+b/τ. This is because, for the renewal model, experimentally plausible ISIs $T_{0}$ are observed only in the vicinity of the bifurcation given by p=b/τ (see Eq. 6). The fact that the fit parameter *a* does not vanish reflects that the model is always poised in the mean-driven regime and thus the parameter pairs systematically fall above the bifurcation line. We find that, even when the ER Ca^2+^ concentration is replaced by its stationary mean value $\left\langle c_{\text{er}}^{*}\right\rangle$, the model still operates in the mean-driven regime, as shown in Fig. 15 *B*.
